# Integrative analysis of the transcriptome and proteome reveals the molecular responses of tobacco to boron deficiency

**DOI:** 10.1186/s12870-024-05391-z

**Published:** 2024-07-19

**Authors:** Jinbin Lin, Xiangli Zheng, Jing Xia, Rongrong Xie, Jingjuan Gao, Rongrong Ye, Tingmin Liang, Mengyu Qu, Yaxin Luo, Yuemin Wang, Yuqin Ke, Chunying Li, Jinping Guo, Jianjun Lu, Weiqi Tang, Wenqing Li, Songbiao Chen

**Affiliations:** 1 Ministerial and Provincial Joint Innovation Centre for Safety Production of Cross-Strait Crops, College of Geography and Oceanography, Minjiang University, Fuzhou, 350108 China; 2 Fujian Key Laboratory of Agricultural Ecological Process of Red Soil Mountain, Agricultural Ecology Institute, Fujian Academy of Agricultural Sciences, Fuzhou, 350013 China; 3 Fujian Institute of Tobacco Sciences, Fuzhou, 350003 China; 4 College of Agriculture, Fujian Agriculture and Forestry University, Fuzhou, 350002 China; 5 College of Plant Protection, Fujian Agriculture and Forestry University, Fuzhou, 350002 China; 6 College of Life Science, Fujian Agriculture and Forestry University, Fuzhou, 350002 China; 7 International Magnesium Institute, Fujian Agriculture and Forestry University, Fuzhou, 350002 China

**Keywords:** Tobacco, Boron, Transcriptome analysis, Proteome analysis, NIP, BOR, Cell wall-related genes, Antioxidant machinery

## Abstract

**Background:**

Boron (B) is an essential micronutrient for plants. Inappropriate B supply detrimentally affects the productivity of numerous crops. Understanding of the molecular responses of plants to different B supply levels would be of significance in crop improvement and cultivation practices to deal with the problem.

**Results:**

We conducted a comprehensive analysis of the transcriptome and proteome of tobacco seedlings to investigate the expression changes of genes/proteins in response to different B supply levels, with a particular focus on B deficiency. The global gene and protein expression profiles revealed the potential mechanisms involved in the responses of tobacco to B deficiency, including up-regulation of the *NIP5;1*-*BORs* module, complex regulation of genes/proteins related to cell wall metabolism, and up-regulation of the antioxidant machinery.

**Conclusion:**

Our results demonstrated that B deficiency caused severe morphological and physiological disorders in tobacco seedlings, and revealed dynamic expression changes of tobacco genes/proteins in response to different B supply levels, especially to B deficiency, thus offering valuable insights into the molecular responses of tobacco to B deficiency.

**Supplementary Information:**

The online version contains supplementary material available at 10.1186/s12870-024-05391-z.

## Background

Boron (B) is an essential micronutrient for plants due to its important role in various physical and metabolic functions. The primary function of B is its involvement in cell wall synthesis and the maintenance of cell wall structure by cross-linking the apiose residues of rhamnogalacturonan II (RG-II) for the formation of pectin complexes which is essential for cell wall structure and function [1]. In addition, B has been proposed to have important roles in maintaining the integrity of plasma membrane [2, 3], regulation of metabolic processes (e.g., hormonal metabolism, nucleic acid synthesis, protein metabolism, carbohydrate metabolism, lignin and flavonoid synthesis, and biosynthesis of antioxidant compounds such as phenols and polyphenols), and regulation of developmental processes (e.g., cell division, growth of the apical meristem, root elongation, stimulation of reproductive tissues, and pollen germination) [4–7].

B homeostasis in plants is regulated by complex processes involved in B uptake, transportation and distribution. The transport of B from the soil into the root and to the shoot is involved the combination of the boric acid channel nodulin 26-like intrinsic proteins (NIPs) (e.g., NIP5;1 which facilitates the uptake of B from the soil into the roots, NIP6;1 and NIP7;1 which implicate in the distribution of B in developing shoot tissues and anthers) [8–10] and B transporters (BORs) (e.g., BOR1 which is responsible for xylem loading of B) [9, 10].

Deficiency and toxicity of B both impede plant growth and development. Morphologically, B deficiency causes inhibition of growing points (e.g., the root tip, bud, flower, and young leaf) and deformities in certain organs (e.g., shoot, leaf, and fruit). For example, B deficiency has been shown to inhibit root growth in *Arabidopsis* [8, 11] and orange [12], reduce leaf growth in rice [13], cause defects in root, vegetative and reproductive development in maize [14], result in abnormal floral organogenesis, and widespread sterility in *Brassica napus* [15], and induce abscission or abortion of reproductive organs in alfalfa [16]. In contrast, B excess causes toxicity in plants. B toxicity impairs photosynthesis, hormone balance, reactive oxygen metabolism, carbohydrate metabolism, nitrogen metabolism, nucleic acid metabolism, cell wall biosynthesis, and leaf and root structure. For example, B toxicity led to leaf necrosis, and inhibition of leaf expansion and root growth in barley [17, 18], inhibition of root growth in *Arabidopsis* [19], and apical leaf mottling and chlorosis in Citrus [20].

Both B deficiency and excess occur worldwide: B deficiency commonly occurs in high rainfall areas with heavily leached soils, for example, Southeast Asia and southeast China [21]; excessive B preferentially occurs in arid and semi-arid areas, for example, South Australia, the Middle East, and many other countries [6]. Since both B deficiency and toxicity result in severe yield losses and quality decline of crops, inappropriate B supply is a widespread problem in agriculture. A deep understanding of the physiological and molecular mechanisms in plants in response to different B supply levels would be of significance in crop improvement and cultivation practices to deal with the problem.

Like many other plants, tobacco is sensitive to deficiency and excess of B [22]. Specifically, B deficiency is prevalent in regions where tobacco is cultivated, while B excess is relatively rare. For example, a study on the spatial distribution of soil available microelements in the Qujing tobacco farming area, Yunan, China, found a widespread deficiency of available B [23]. Several studies have reported physiological and molecular responses of tobacco to B deficiency, e.g., decline in nitrate content and nitrate reductase (NR) activity, increment in carbohydrate content, accumulation of chlorogenic acid, caffeoyl polyamine conjugates and phenylpropanoids, increment in phenylalanine ammonia-lyase (PAL) and polyphenoloxidase (PPO) activities in leaves [24–26], increment in putrescine levels in tobacco plants [27], and up-regulation of an asparagine synthetase (*AS*) gene and the glutamate dehydrogenase (*GDH*) genes in roots [28, 29]. However, our understanding of the general responses of tobacco to different B supply levels remains limited. In the present study, we conducted a comprehensive analysis of the transcriptome and proteome of tobacco seedlings to investigate the expression changes of genes and proteins associated with different B supply levels, with a particular focus on B deficiency. Our findings contribute to a deeper understanding of the molecular mechanism underlying the responses of tobacco to B deficiency.

## Results

### B deficiency causes severe morphological and physiological disorders in tobacco seedlings

**Fig. 1:**
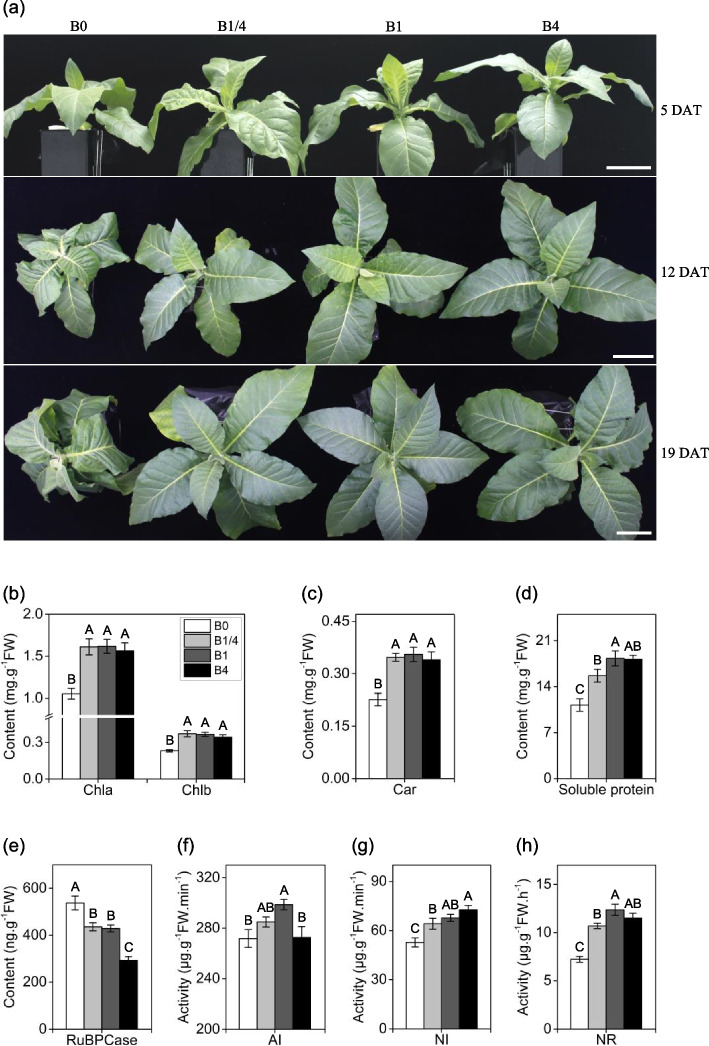
Effects of different B supplies on growth and physiological parameters of tobacco seedlings. **a** Phenotype of tobacco seedlings grown under different B supply levels at 5, 12 and 19 DAT (days after treatment), respectively. **b**, **c** Contents of chlorophyll a (Chla), chlorophyll b (Chlb), and carotenoids (Car), respectively, of tobacco seedlings grown under different B supplies at 19 DAT. **d** Concentration of soluble proteins (SP) of tobacco seedlings grown under different B supplies at 19 DAT. (**e**) Content of RuBPCase of tobacco seedlings grown under different B supplies at 19 DAT. (**f**, **g**, **h**) Activities of acid invertase (AI), neutral invertase (NI), and nitrate reductase (NR), respectively, of tobacco seedlings grown under different B supplies at 19 DAT. B0, 0 μM B; B1/4, 11.57 μM B; B1, 46.26 μM B; B4, 185.04 μM B. Different letters (A, B, C, D) above the columns indicate statistical differences (*p* < 0.01)

Tobacco seedlings were cultured in modified Hoagland nutrient solution with a B concentration of 46.26 μM [30] (designated as B1), and in modified Hoagland nutrient solutions containing three B levels (0, 11.57 and 185.04 μM, designated as B0, B1/4, and B4, respectively). At five days after treatment (DAT), the B1/4, B1 and B4 seedlings exhibited no obvious differences in morphology, whereas the B0 seedlings displayed a slightly dwarf phenotype (Fig. 1a). At 12 and 19 DAT, the B0 seedlings displayed severe symptoms (e.g., growth stunting, leaf curling and plant deformity) (Fig. 1a). In contrast, the B1/4, B1 and B4 seedlings exhibited no obvious differences in morphology (Fig. 1a).

Tobacco seedlings grown under different B concentrations at 19 DAT were measured for eight physiological traits related to photosynthesis, carbon and nitrogen metabolisms. The levels of three essential photosynthetic pigments, namely chlorophyll a (Chla), chlorophyll b (Chlb), and carotenoids (Car), as well as the soluble protein (SP) regarded as the important osmoregulatory substance, were significantly reduced in the B0 seedlings compared with that in the B1/4, B1 and B4 seedlings (Fig. 1b-d). The activity of RuBPCase which is a key C3 enzyme responsible for carbon fixation was significantly increased in the B0 seedlings compared with that in the B1/4, B1 and B4 seedlings (Fig. 1e). In contrast, the activities of acid invertase (AI) and neutral invertase (NI) both of which are involved in carbon metabolism, and NR, a key enzyme in nitrogen metabolism, were markedly reduced in the B0 seedlings compared with that in the B1/4, B1 and B4 seedlings (Fig. 1f-h). Collectively, these results demonstrated that a deficiency of B causes severe morphological and physiological disorders in tobacco seedlings.

### Profiling of genes/proteins differentially expressed in response to different B supply levels

The leaves of tobacco seedlings grown under various B levels at 5, 12, and 19 DAT, respectively, were collected for RNA-seq analysis. Additionally, the leaf samples collected at 12 DAT were subjected to proteome analysis.

**Fig. 2:**
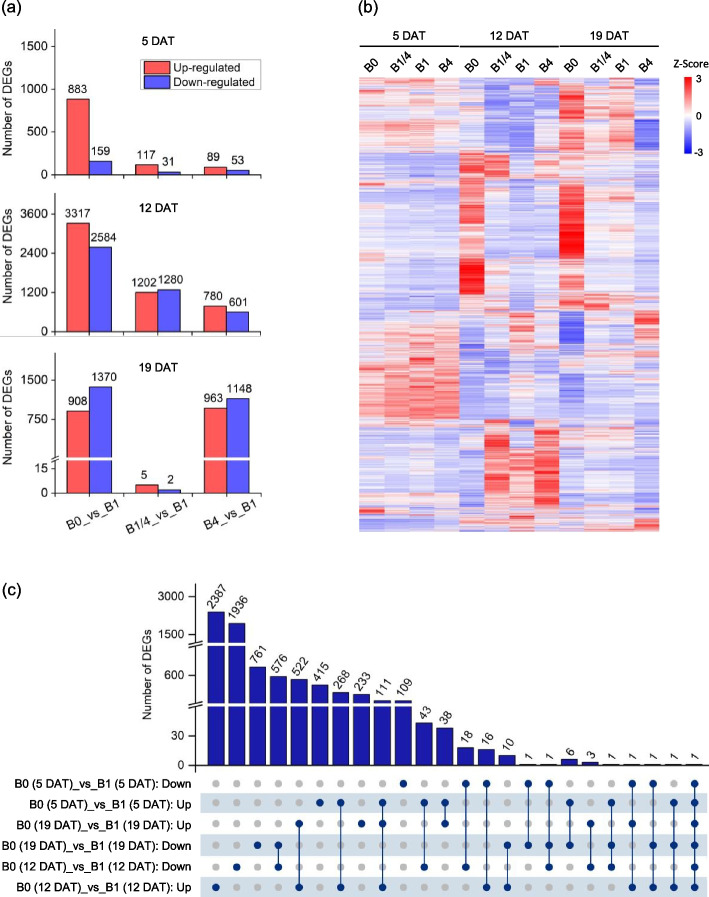
Overview of the differentially expressed genes (DEGs) in response to different B supplies in tobacco seedlings. **a** Numbers of the identified DEGs of tobacco seedlings grown under different B supplies at 5, 12 and 19 DAT, respectively. **b** Heatmap showing the differential expression levels of the identified DEGs in response to different B supplies at 5, 12 and 19 DAT, respectively. **c** Upset diagram showing the numbers of the DEGs specific to or common to different B supplies at 5, 12 or 19 DAT, respectively. B0, 0 μM B; B1/4, 11.57 μM B; B1, 46.26 μM B; B4, 185.04 μM B

A total of 10,311 genes were identified that were differentially expressed in the B0, B1/4, or B4 seedlings in comparison to the B1 seedlings (Fig. 2, Table S1, Table S2). At 5 and 12 DAT, the B0 seedlings exhibited a higher number of differentially expressed genes (DEGs) compared to the B1/4 and B4 seedlings. At 19 DAT, the B0 and B4 seedlings demonstrated a greater number of DEGs compared to the B1/4 seedlings (Fig. 2a-b). These results indicated that B deficiency induces substantial transcriptomic changes in tobacco seedlings, while over-sufficient B level could also lead to significant transcriptomic changes over an extended treatment duration. An upset analysis was performed on the DEGs obtained from the comparison between B0 and B1 to gain more information about the regulation patterns of the DEGs in response to B deficiency (Fig. 2c). About 522 DEGs were co-up-regulated at both 12 DAT and 19 DAT, and 576 DEGs were co-down-regulated at both 12 DAT and 19 DAT. Notably, 111 DEGs were up-regulated at all three time points (Fig. 2c). These results suggested that a significant number of DEGs exhibited relatively long-term regulation in tobacco seedlings in response to B deficiency.

**Fig. 3:**
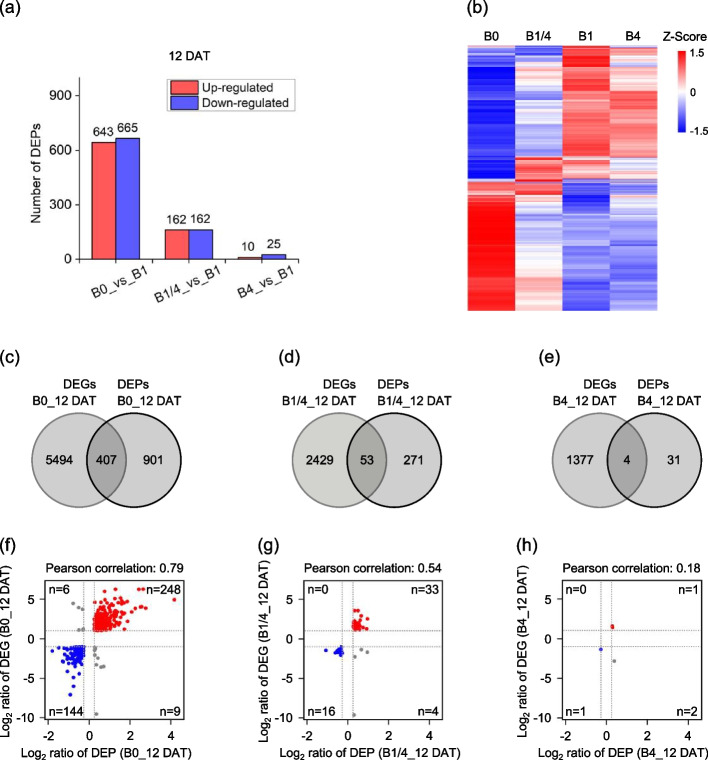
Overview of the differentially expressed proteins (DEPs) in response to different B supplies. **a** Number of the identified DEPs of tobacco seedlings grown under different B supplies at 12 DAT. **b** Heatmap showing the differential expression levels of the identified DEPs in response to different B supplies at 12 DAT. **c**, **d**, **e** Venn diagrams showing the numbers of associated DEGs-DEPs identified in the B0, B1/4, and B4 seedlings at 12 DAT, respectively. **f**, **g, h** Correlation between the DEGs and DEPs in the B0, B1/4, and B4 seedlings at 12 DAT, respectively. The number of associated DEGs-DEPs (n) is indicated within each quadrant. B0, 0 μM B; B1/4, 11.57 μM B; B1, 46.26 μM B; B4, 185.04 μM B

A total of 1,416 proteins were identified that were differentially expressed in the B0, B1/4, or B4 seedlings in comparison to the B1 seedlings at 12 DAT (Fig. 3, Table S3, Table S4). Consistent with the profiling results of DEGs in response to varying B supply levels, a higher number of differentially expressed proteins (DEPs) were observed in the B0 seedlings compared to the B1/4 and B4 seedlings (Fig. 3a-b), indicating that B deficiency induces significant proteomic changes in tobacco seedlings. Among the identified DEPs, 643, 162, and 10 were up-regulated in the B0, B1/4, and B4 seedlings, respectively; and 665, 162, and 25 were down-regulated in the B0, B1/4, and B4 seedlings, respectively (Fig. 3a).

The DEPs and DEGs identified in the B0, B1/4, and B4 seedlings in comparison to the B1 seedlings at 12 DAT were compared, and approximately 407, 53 and 4 DEPs were among the DEGs identified in the B0, B1/4, and B4 seedlings, respectively (Fig. 3c-e). Among the 407 associated DEGs-DEPs identified in the B0 seedling, 248 were co-up-regulated and 144 were co-down-regulated (Fig. 3f). Similarly, the majority of the associated DEGs-DEPs identified in the B1/4 or B4 seedlings showed positively correlated regulation patterns (Fig. 3g-h). Overall, the integrated transcriptome and proteome analysis revealed dynamic gene expression changes in tobacco seedlings in response to different B supply levels.

### Functional classification of DEGs/DEPs in response to B deficiency by GO analysis

**Fig. 4:**
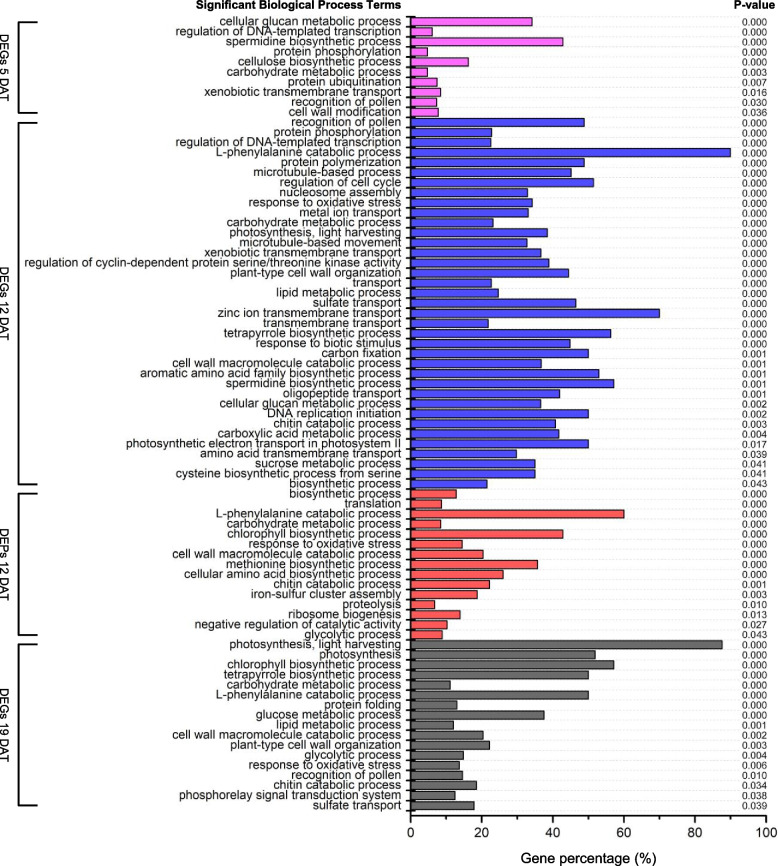
Significant Gene Ontology (GO) biological process (BP) terms of the DEGs/DEPs identified from the comparison between the B0 and B1 seedlings at 5, 12 or 19 DAT, respectively

The DEGs and DEPs identified from the comparison between the B0 and B1 seedlings were subjected to gene ontology (GO) analysis. Since GO biological process (BP) depicts biological goals accomplished by a collection of molecular events, we thus looked into the detailed information of the significant BP terms. The results showed that the DEGs/DEPs were enriched in diverse BP terms (Fig. 4, Table S5). Notably, the DEGs at 5 DAT, DEGs/DEPs at 12 DAT, and DEGs at 19 DAT were significantly enriched in several BP terms related to transport processes, cell wall processes, and antioxidative processes. For instance, the DEGs at 5 DAT were significantly enriched in one term related to transport processes (xenobiotic transmembrane transport) and two terms related to cell wall processes (cellulose biosynthetic process and cell wall modification); the DEGs at 12 DAT were significantly enriched in eight terms related to transport processes (metal ion transport, xenobiotic transmembrane transport, transport, sulfate transport, zinc ion transmembrane transport, transmembrane transport, oligopeptide transport, and amino acid transmembrane transport), three terms related to cell wall processes (plant-type cell wall organization, cell wall macromolecule catabolic process, and chitin catabolic process), and one term related to antioxidative processes (response to oxidative stress); the DEPs at 12 DAT were significantly enriched in two terms related to cell wall processes (e.g., cell wall macromolecule catabolic process and chitin catabolic process) and one term related to antioxidative processes (response to oxidative stress); and the DEGs at 19 DAT were significantly enriched in three terms related to cell wall processes (e.g., cell wall macromolecule catabolic process, plant-type cell wall organization, and chitin catabolic process), one term related to antioxidative processes (response to oxidative stress), and one term related to transport processes (sulfate transport) (Fig. 4).

### Expressional regulation of DEGs involved in B uptake and distribution

The GO analysis results indicated that the expression of numerous genes related to transport processes, cell wall processes, and antioxidative processes were differentially affected by B deficiency. Therefore, we characterized the DEGs/DEPs involved in these three aspects.

**Fig. 5:**
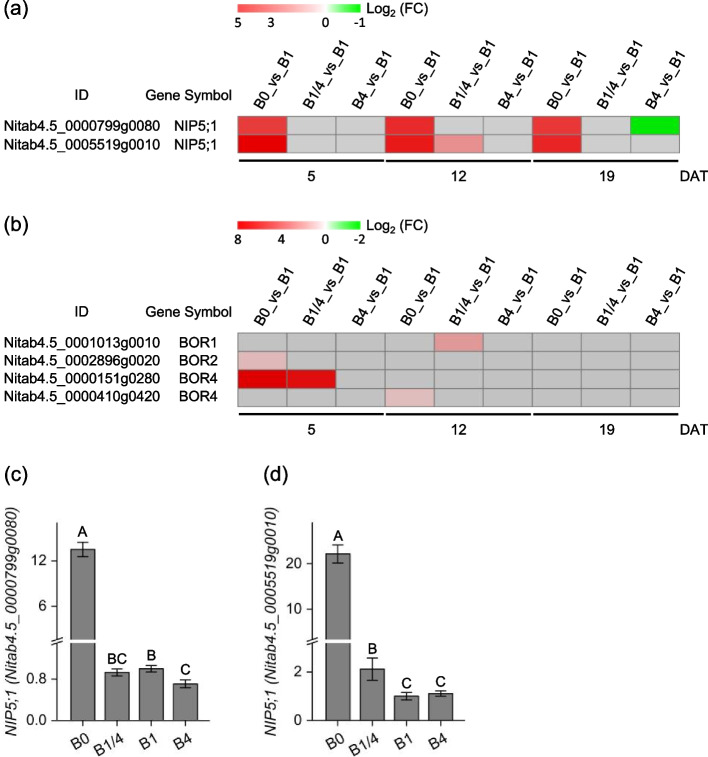
Expressional changes of key DEGs involved in B uptake and distribution in tobacco in response to different B supplies. **a**, **b** Heatmap showing the differential expression levels of two Nodulin-26-like intrinsic protein 5;1 (NIP5;1) DEGs and four B transporters (BORs) DEGs, respectively, at 5, 12 and 19 DAT. Grey blocks indicate that the genes were not detected as DEGs by RNA-seq. **c**, **d** qRT-PCR validation of expression profiles of two *NIP5;1* (*Nitab4.5_0000799g0080* and *Nitab4.5_0005519g0010*) in the B0, B1/4, B1, and B4 seedlings, respectively, at 12 DAT. B0, 0 μM B; B1/4, 11.57 μM B; B1, 46.26 μM B; B4, 185.04 μM B. Different letters (A, B, C) above the columns indicate statistical differences (*p* < 0.01)

Nodulin-26-like intrinsic protein 5;1 (NIP5;1) functions as a boric acid channel responsible for the B cellular uptake [8]. Two *NIP5;1* genes (*Nitab4.5_0000799g0080* and *Nitab4.5_0005519g0010*) were identified among the DEGs, and both were greatly up-regulated in the B0 seedlings at 5, 12, and 19 DAT (Fig. 5a). The transcriptional profiles of the two *NIP5;1* genes in the B0, B1/4, B1, and B4 seedlings, respectively, at 12 DAT were validated by real-time RT-PCR (Fig. 5c-d), and the results were consistent with RNA-seq data. B transporters (BORs) are responsible for B distribution through transferring B to neighboring cell types or to the apoplast [31]. Four *BORs* were identified among the DEGs, including one *BOR1* (*Nitab4.5_0001013g0010*), one *BOR2* (*Nitab4.5_0002896g0020*), and two *BOR4* (*Nitab4.5_0000151g0280*, and *Nitab4.5_0000410g0420*). The expression of all four *BORs* was up-regulated in the B0 or B1 seedlings at 5 or 12 DAT (Fig. 5b).

### Expressional regulation of DEGs/DEPs involved in cell wall metabolism

**Fig. 6:**
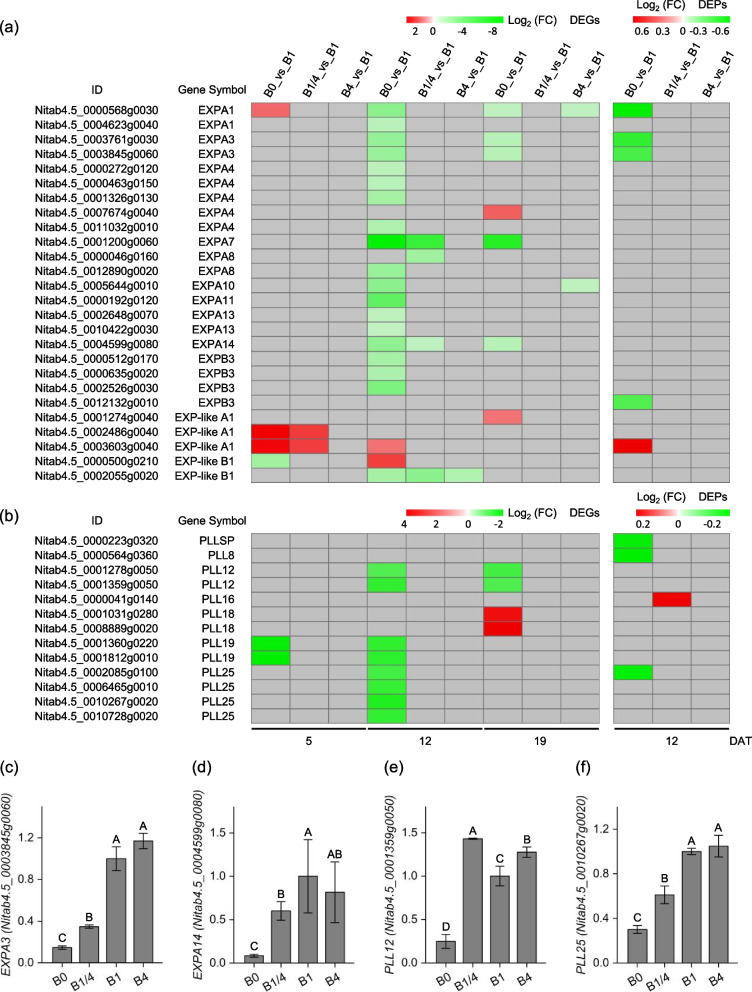
Expressional changes of the expansin (EXP) family and the pectate lyase-like protein (PLL) family DEGs/DEPs in response to different B supplies. **a**, **b** Heatmap showing the differential expression levels of the EXP family and the PLL family DEGs/DEPs, respectively, at 5, 12 and 19 DAT. Grey blocks indicate that the genes were not detected as DEGs or DEPs by RNA-seq or TMT-based quantitative proteome analysis. **c**, **d**, **e**, **f** qRT-PCR validation of expression profiles of the DEGs *EXPA3* (*Nitab4.5_0003845g0060*), *EXPA14* (*Nitab4.5_0004599g0080*), *PLL12* (*Nitab4.5_0001359g0050*), and *PLL25* (*Nitab4.5_0010267g0020*) in the B0, B1/4, B1, and B4 seedlings, respectively, at 12 DAT. B0, 0 μM B; B1/4, 11.57 μM B; B1, 46.26 μM B; B4, 185.04 μM B. Different letters (A, B, C) above the columns indicate statistical differences (*p* < 0.01)

Expansins (EXPs) are plant cell wall proteins that play important roles in cell loosening [32]. A total of 26 EXPs were identified among the DEGs/DEPs. Among them, while one EXPA4 (Nitab4.5_0007674g0040), three EXP-like A1 (Nitab4.5_0001274g0040, Nitab4.5_0002486g0040, and Nitab4.5_0003603g0040), and one EXP-like B1 (Nitab4.5_0000500g0210) were up-regulated in the B0 or B1/4 seedlings at 5, 12 and/or 19 DAT, the majority of the EXP DEGs/DEPs were significantly down-regulated in response to B deficiency at 12 and/or 19 DAT (Fig. 6a). One EXPA1 (Nitab4.5_0000568g0030) was up-regulated in the B0 seedlings at 5 DAT, but was down-regulated in the B0 seedlings at 12 and 19 DAT (Fig. 6a). Pectate lyase-like proteins (PLLs) are enzymes involved in plant cell wall degradation cleaving pectin molecules [33]. About 13 PPLs were identified among the DEGs/DEPs. Similar to the regulation patterns of the differentially expressed EXPs, while one DEP of PLL16 homolog (Nitab4.5_0000041g0140) was up-regulated in the B1/4 seedlings at 12 DAT, and two DEGs encoding PLL18 homolog (*Nitab4.5_0001031g0280* and *Nitab4.5_0008889g0020*) were up-regulated in the B0 seedlings at 19 DAT, the remaining 10 PPLs were significantly down-regulated in response to B deficiency at 5, 12 and/or 19 DAT (Fig. 6b). The transcriptional profiles of four DEGs *EXPA3* (*Nitab4.5_0003845g0060*), *EXPA14* (*Nitab4.5_0004599g0080*), *PLL12* (*Nitab4.5_0001359g0050*), and *PLL25* (*Nitab4.5_0010267g0020*) in the B0, B1/4, B1, and B4 seedlings, respectively, at 12 DAT were validated by real-time RT-PCR (Fig. 6c-f), and the results were consistent with RNA-seq data.

**Fig. 7:**
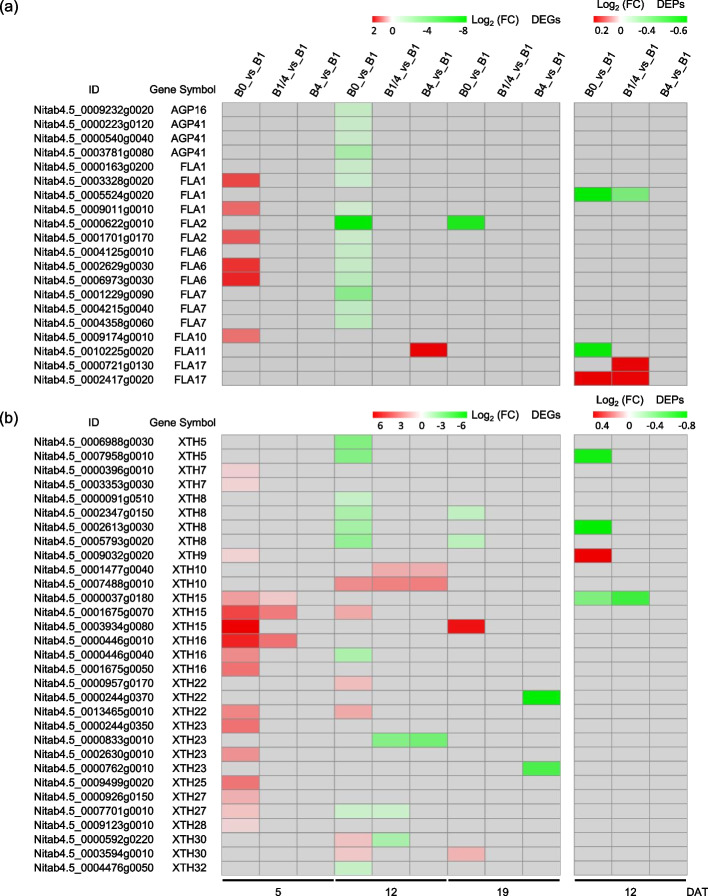
Expressional changes of the arabinogalactan protein/fasciclin-like arabinogalactan protein (AGP/FLA) family and the xyloglucan endotransglucosylases/hydrolase (XTH) family DEGs/DEPs in response to different B supplies. **a**, **b** Heatmap showing the differential expression levels of the AGP/FLA family and the XTH family DEGs/DEPs, respectively, at 5, 12 and 19 DAT. Grey blocks indicate that the genes were not detected as DEGs or DEPs by RNA-seq or TMT-based quantitative proteome analysis. B0, 0 μM B; B1/4, 11.57 μM B; B1, 46.26 μM B; B4, 185.04 μM B

Arabinogalactan proteins (AGPs) are a family of hydroxyproline-rich glycoproteins that are abundant in the plant cell wall and plasma membrane, and they are believed to play important roles in modulating cell wall mechanics [34, 35]. Fasciclin-like arabinogalactan proteins (FLAs) are a subclass of AGPs involved in cell adhesion [36]. Four AGP family genes were identified among the DEGs, including one *AGP16* (*Nitab4.5_0009232g0020*), and three *AGP41* (*Nitab4.5_0000223g0120*, *Nitab4.5_0000540g0040*, and *Nitab4.5_0003781g0080*). All four *AGPs* were down-regulated in the B0 seedlings at 12 DAT (Fig. 7a). Sixteen FLAs were identified among the DEGs/DEPs. Among them, six *FLAs* identified as DEGs at 5 DAT were all up-regulated in response to B deficiency (Fig. 7a). In contrast, 13 out of 15 FLAs identified as DEGs/DEPs at 12 DAT were found to be down-regulated in the B0 seedlings, except for two DEPs of FLA17 homologs Nitab4.5_0000721g0130 and Nitab4.5_0002417g0020, which were up-regulated in the B0 and/or B1/4 seedlings (Fig. 7a). Among these identified *FLAs*, five (*FLA1/Nitab4.5_0003328g0020*, *FLA1/Nitab4.5_0009011g0010*, *FLA2/Nitab4.5_0001701g0170*, *FLA6/Nitab4.5_0002629g0030*, and *FLA6 Nitab4.5_0006973g0030*) were found to be up-regulated at 5 DAT but down-regulated at 12 DAT in response to B deficiency (Fig. 7a).

Xyloglucan endotransglucosylases/hydrolases (XTHs) are enzymes involved in rearranging the association network of xyloglucans and cellulose microfibrils [37], playing a pivotal role in cell wall relaxation [38]. A total of 31 XTHs were identified among the DEGs/DEPs. Similar to the regulation patterns observed for the differentially expressed *XTHs* identified at 5 DAT, 16 *XTHs* identified as DEGs at 5 DAT were all up-regulated in response to B deficiency (Fig. 7b). At 12 and/or 19 DAT, 22 XTHs were identified as DEGs/DEPs but did not exhibit synergistic regulatory patterns. Among them, eight were up-regulated, and 10 were down-regulated in the B0 seedlings (Fig. 7b). Five *XTHs* (*XTH9/Nitab4.5_0009032g0020, XTH15/Nitab4.5_0001675g0070*, *XTH/Nitab4.5_0003934g0080*, *XTH22/Nitab4.5_0013465g0010*, and *XTH30/Nitab4.5_0003594g0010*) shown continuous up-regulation, while two *XTH8* (*Nitab4.5_0002347g0150* and *Nitab4.5_0005793g0020*) exhibited continuous down-regulation in the B0 seedlings at different time points.

Pectin methylesterases (PMEs) and pectin methylesterase inhibitors (PMEIs) are two families of enzymes that counteract each other and play critical roles in modifying pectins, a type of polysaccharides in the plant cell walls [39, 40]. A total of 29 PMEs and 20 pectinesterase inhibitor domain-containing genes/proteins, including nine PMEIs, four cell wall/vacuolar inhibitors of fructosidases (C/VIFs) and seven plant invertase/pectin methylesterase inhibitor superfamily proteins (INV/PMEI) were identified among the DEGs/DEPs (Fig. S1). While five *PMEs* (*PME1/Nitab4.5_0008337g0040*, *PME12/Nitab4.5_0000905g0050*, *PME12/Nitab4.5_0004278g0010*, *PMEI-PME18/Nitab4.5_0010008g0010*, and *PMEI-PME51/Nitab4.5_0000805g0010*) and one *PMEI* (*PMEI9/Nitab4.5_0004482g0040*) were synergistically up-regulated at 5 DAT in the B0 seedlings, the DEGs/DEPs identified at 12 and/or 19 DAT did not exhibit coordinated regulatory patterns (Fig. S1). Overall, these findings indicated that B deficiency led to differential expression of numerous genes/proteins involved in cell wall metabolism in complex regulatory patterns.

### Expressional regulation of DEGs/DEPs involved in antioxidant responses

**Fig. 8:**
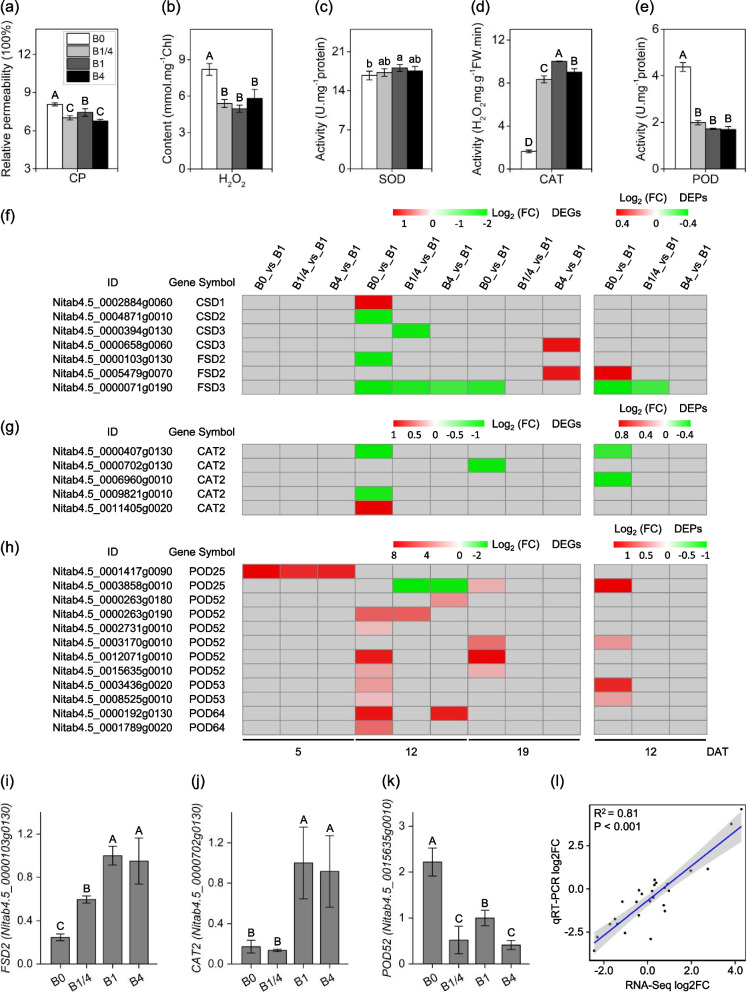
Effects of different B supplies on physiological parameters related to antioxidant defense and expressional changes of key DEGs/DEPs involved in enzymatic scavenging in response to different B supplies. **a**, **b** Relative cell membrane permeability (CP) and hydrogen peroxide (H_2_O_2_) content, respectively, of tobacco seedlings grown under different B supplies at 19 DAT. **c**, **d**, **e**) Activities of superoxide dismutase (SOD), catalase (CAT) and peroxidase (POD) of tobacco seedlings grown under different B supplies at 19 DAT. **f**, **g**, **h** Heatmap showing the differential expression levels of the superoxide dismutase (SOD) family, the catalase family (CAT2) and the peroxidase (POD) family DEGs/DEPs, respectively, at 5, 12 and 19 DAT. Grey blocks indicate that the genes were not detected as DEGs or DEPs by RNA-seq or TMT-based quantitative proteome analysis. (i, j, k) qRT-PCR validation of expression profiles of the DEGs *FSD2* (*Nitab4.5_0000103g0130*), *CAT2* (*Nitab4.5_0000702g0130*), and *POD52* (*Nitab4.5_0015635g0010*) in the B0, B1/4, B1, and B4 seedlings at 12, 19, and 12 DAT, respectively. (l) Significant correlation between RNA-seq and qRT-PCR for nine DEGs (*NIP5;1/Nitab4.5_0000799g0080*, *NIP5;1/Nitab4.5_0005519g0010*, *EXPA3/Nitab4.5_0003845g0060*, *EXPA14/Nitab4.5_0004599g0080*, *PLL12/Nitab4.5_0001359g0050*, *PLL25/Nitab4.5_0010267g0020*, *FSD2/Nitab4.5_0000103g0130*, *CAT2/Nitab4.5_0000702g0130*, and *POD52/Nitab4.5_0015635g0010*) indicated by Pearson’s correlation. B0, 0 μM B; B1/4, 11.57 μM B; B1, 46.26 μM B; B4, 185.04 μM B. Different letters (A, B, C, D) or (a, b, c, d) above the columns indicate statistical differences (*p* < 0.01) or (*p* < 0.05), respectively

The physiological traits related to antioxidant responses were measured in tobacco seedlings grown under different B supplies at 19 DAT (Fig. 8a-e). The relative levels of cell membrane permeability (CP) (Fig. 8a) and H_2_O_2_ content (Fig. 8b) in B0 were found to be significantly higher than that in the B1/4, B1, and B4 seedlings, suggesting the occurrence of oxidative stress in tobacco seedlings in response to B deficiency.

Superoxide dismutases (SODs) are enzymes that catalyze the dismutation of oxide ions (O^2−^) into hydrogen peroxide (H_2_O_2_). The activity of SOD in B0 was slightly lower than or comparable to that in the B1/4, B1, and B4 seedlings (Fig. 8c). Among the DEGs/DEPs, seven SODs were identified, comprising of four copper/zinc superoxide dismutases (CSDs) and three iron superoxide dismutases (FSDs). Two *SOD* DEGs/DEPs (*CSD1/Nitab4.5_0002884g0060* and FSD2/Nitab4.5_0005479g0070) were found to be up-regulated in the B0 seedlings at 12 DAT, while three SOD DEGs/DEPs (*CSD2/Nitab4.5_0004871g0010*, *FSD2/Nitab4.5_0000103g0130*, and *FSD3/Nitab4.5_0000071g0190*) were down-regulated in the B0 seedlings at 12, and/or 19 DAT (Fig. 8f). Catalases (CAT2s) and peroxidases (PODs) are enzymes responsible for the conversion of H_2_O_2_ into water. In response to B deficiency, the activities of CAT and POD exhibited contrasting regulation patterns: CAT activity significantly decreased (Fig. 8d), whereas POD activity significantly increased in B0 seedlings (Fig. 8e). Five CAT2s were identified among the DEGs/DEPs. The majority of the CAT2 DEGs/DEPs (four out of five) was found to be down-regulated in the B0 seedlings at 12 and/or 19 DAT (Fig. 8g). Among the DEGs/DEPs, approximately 12 PODs were identified, with 11 out of 12 being up-regulated in B0 seedlings at 5, 12, and/or 19 DAT (Fig. 8h). The transcriptional profiles of the DEGs *FSD2* (*Nitab4.5_0000103g0130*), *CAT2* (*Nitab4.5_0000702g0130*), and *POD52* (*Nitab4.5_0015635g0010*) in the B0, B1/4, B1, and B4 seedlings at 12, 19, and 12 DAT, respectively, were validated by real-time RT-PCR (Fig. 8i-k), and the results were consistent with RNA-seq data. The log2 fold changes in the expression levels of nine DEGs (*NIP5;1/Nitab4.5_0000799g0080*, *NIP5;1/Nitab4.5_0005519g0010*, *EXPA3/Nitab4.5_0003845g0060*, *EXPA14/Nitab4.5_0004599g0080*, *PLL12/Nitab4.5_0001359g0050*, *PLL25/Nitab4.5_0010267g0020*, *FSD2/Nitab4.5_0000103g0130*, *CAT2/Nitab4.5_0000702g0130*, and *POD52/Nitab4.5_0015635g0010*) detected by RNA-seq and qRT-PCR exhibited a significant correlation (*R*^2^ = 0.81, *P* < 0.001) (Fig. 8l). The regulation patterns of the majority of the CAT2 and POD DEGs/DEPs were consistent with the decrease of CAT activity and enhancement of POD activity, respectively, in tobacco seedlings grown under B deficiency.

Glutathione S-transferases (GSTs) are enzymes that protect cells from oxidative stress by quenching reactive molecules through the addition of glutathione (GSH) [41]. Among the DEGs/DEPs, a total of 33 GST family genes were identified (Fig. S2). In tobacco seedlings grown under B deficiency at 5, 12, and/or 19 DAT, 31 GST DEGs/DEPs were found to be up-regulated, except for *GSTU8/Nitab4.5_0008696g0020* and *GSTF8/Nitab4.5_0010451g0030*, which were down-regulated in the B1/4, and B0 seedlings, respectively, at 12 DAT (Fig. S2).

## Discussion

B is an essential micronutrient for the growth and development of plants. Inappropriate supply of B detrimentally affects the productivity of numerous crops. In the present study, we conducted transcriptome and proteome analyses to investigate the molecular responses of tobacco to varying levels of B supply. The analyses demonstrated dynamic changes in the expression of tobacco genes/proteins in response to varying levels of B supply, particularly under B deficiency conditions.

Two primary types of transporters, namely *NIPs* (facilitating B permeation) and *BORs* (facilitating B export), have been identified to be involved in B uptake and transport [42]. Among the NIPs, NIP5;1 has been demonstrated as a major boric acid channel that efficiently facilitated the uptake of extracellular B into the cell [8, 43]. The mutation or knockdown of *AtNIP5;1* or its homologs, has been found to induce severe B deficiency symptoms in *Arabidopsis* [8], *Brassica napus* [44], maize [14, 45], and rice [46, 47]. The tobacco genome contains two genes belonging to the *NIP5;1* family [48]. In the present study, both the two *NIP5;1* genes were identified as DEGs that exhibited up-regulated under B deficiency (Fig. 5a), consistent with the findings of previous studies in *Arabidopsis* [8], rice [46], maize [14], *Medicago truncatula* [49], *B. napus* [50], sugar beet [51], and pear [52], thereby suggesting that the two *NIP5;1* genes should be the key components responsible for B uptake in tobacco.

**Fig. 9:**
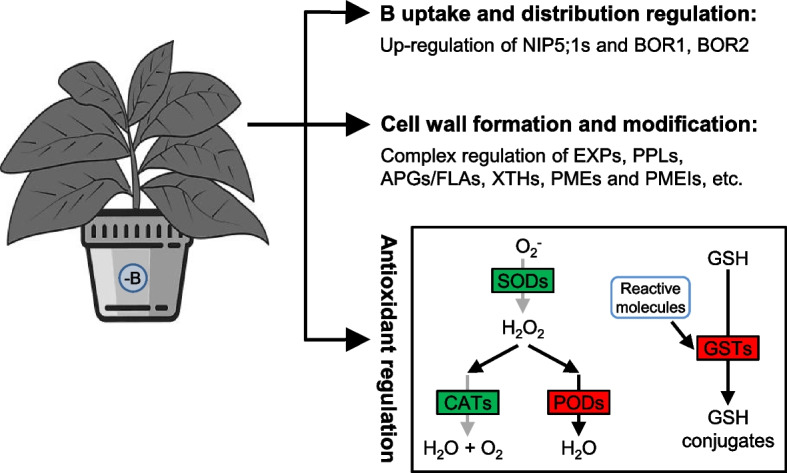
Schematic model of the molecular regulation mechanisms of B uptake and distribution, cell wall formation and modification, and antioxidant machinery underlying the responses in tobacco to B deficiency. NIP5;1, nodulin-26-like intrinsic protein 5;1; BOR, B transporter; EXPs, expansins; PPLs, pectate lyase-like proteins; APGs/FLAs, arabinogalactan proteins/asciclin-like arabinogalactan proteins; XTHs, xyloglucan endotransglucosylases/hydrolases; PMEs, pectin methylesterases; PMEIs, pectin methylesterase inhibitors; SODs, superoxide dismutases; CATs, catalases; PODs, peroxidases; GSH, glutathione; GSTs, glutathione S-transferases. Red and green backgrounds indicate that the genes/proteins were up-regulated and down-regulated, respectively

Four *BORs* were identified as DEGs, including one *BOR1* homolog, one *BOR2* homolog, and two *BOR4* homologs. These genes were induced in tobacco seedlings under B deficiency or relative limitation at 5 DAT or 12 DAT (Fig. 5b). Previous studies have shown that *BOR1* encodes an efflux-type B transporter that is required for efficient B uptake, xylem loading of B, and is involved in the preferential distribution of B to young leaves [13, 53, 54]. *BOR2* has been reported to function as an efflux-type B transporter and, under B-limited conditions, facilitate the transport of B to the apoplast for effective cross-linking of pectic polysaccharide rhamnogalacturonan-II (RG-II) in cell wall [55]. Therefore, up-regulation of *BOR1* and *BOR2* under B-deficient/limiting conditions should be beneficial for maintaining B homeostasis in the leaves of tobacco seedlings (Fig. 9). Unlike *BOR1* and *BOR2*, which are required under low-B conditions, *BOR4* has been identified to play a role in excluding B to counteract B toxicity [56]. In the present study, it seemed paradoxical that the two *BOR4* homologs were up-regulated under B-deficient/limiting conditions (Fig. 5b). Whether these *BOR4s* are involved in B uptake and distribution in tobacco remains to be further explored.

It widely acknowledged that one of the primary functions of B in plants is its structural role in the cell wall [42]. The plant cell wall is composed of cellulose, hemicellulose, pectin, lignin and cell wall protein [57]. B has a key function in cross-linking pectin chains, which is essential for maintaining cell wall structure and function through the formation of borate esters with apiose residues of RG-II [1]. B deficiency has been found to result in abnormal cell wall structure and severe symptoms associated with cell wall formation and modification (e.g., cessation of root elongation and curling of leaves) [58, 59]. B deficiency affected the expression levels of genes/proteins related to cell wall metabolism. For example, previous studies have shown the down-regulation of multiple cell wall-related genes in the roots of *Arabidopsis* [60], the up-regulation of 18 proteins involved in cell wall metabolism in the roots of *Citrus sinensis* [61], the highly regulation (either down-regulation or up-regulation) of 13 genes involved in cell wall metabolism in Citrus rootstock roots [62], and the up-regulation of several genes involved in cell wall metabolism in shoot apices of pea plants in response to B deficiency [16]. In consistent with previous studies, in the present study, a large number of cell wall-related genes/proteins were significantly regulated in response to different B supply levels, including EXPs, PPLs, APGs/FLAs, XTHs, PMEs and PMEIs. Similarly, these cell wall-related genes/proteins did not exhibit synergistic regulatory patterns: the majority of the EXP family DEGs/DEPs were down-regulated in response to B deficiency at 12 and/or 19 DAT (Fig. 6a); the majority of the PLL family DEGs/DEPs were down-regulated in response to B deficiency at 5 and/or 12 DAT (Fig. 6b); the majority of the APG/FLA and XTH family DEGs/DEPs were up-regulated in response to B deficiency at 5 DAT, but were down-regulated or did not exhibit synergistic regulatory patterns at 12 and/or 19 DAT, respectively (Fig. 7a, b); and the PME and PMEI family DEGs/DEPs did not exhibit synergistic regulatory patterns in response to B deficiency (Fig. S1). These results demonstrated the pivotal influence of B deficiency on plant cell wall formation and modification, and suggested a complex mechanism involving in expression regulation of cell wall-related genes/proteins in response to B deficiency.

Plants are prone to oxidative stress due to micronutrient deficiencies [63, 64]. The level of cell membrane damage (indicated by CP) and the H_2_O_2_ content (Fig. 8a-b) were notably enhanced in tobacco seedlings experiencing B deficiency, indicating that B deficiency induced oxidative stress in tobacco. Plants regulate oxidative stress through enzymatic and non-enzymatic systems [65]. The enzymatic system is mainly involved in SOD, CAT, POD and GST [30]. Surprisingly, it was observed that the activities of SOD and CAT decreased in tobacco seedlings in response to B deficiency (Fig. 8c-d), and the majority of the *SOD* family and *CAT2* family DEGs/DEPs were down-regulated in response to B deficiency at 12 and/or 19 DAT (Fig. 8f-g), further supporting the measurement results of SOD and CAT activities. The decreases in SOD and CAT activities could be related to the metabolic disorder due to B deficiency or other unknown reasons. In contrast, the activity of POD was significantly increased in tobacco seedlings under B deficiency conditions (Fig. 8c). Correspondingly, the majority of the POD family DEGs/DEPs were up-regulated in response to B deficiency (Fig. 8h). A large number of 33 GST family DEGs/DEPs were also identified in tobacco seedlings under B deficiency. Notably, 31 out 33 identified GST DEGs/DEPs were up-regulated in response to B deficiency (Fig. S2). The increase in POD activity, along with the up-regulation pattern of observed in numerous POD and GST DEGs/DEPs indicated the activation of the antioxidant defense system in tobacco to mitigate oxidative stress caused by B deficiency (Fig. 8).

## Conclusion

Combined transcriptome and proteome analysis revealed dynamic expression changes of tobacco genes/proteins in response to different B supply levels, especially to B deficiency. The global gene and protein expression profiles revealed the potential mechanisms involved in the responses of tobacco to B deficiency. These mechanisms include up-regulation of the *NIP5;1*-*BORs* module, complex regulation of genes/proteins related to cell wall metabolism, and up-regulation of the antioxidant machinery. Our results offer valuable insights into the molecular responses of tobacco to B deficiency.

## Methods

### Plant materials and growth conditions

Seeds of tobacco (*Nicotiana tabacum* L. cv. CB-1) (maintained in Fujian Institute of Tobacco Sciences) were placed in soil trays, and the germinated seedlings were grown under a condition of 12-h light, 25 °C/12-h dark, 20 °C till 7 leaves. The well-growth tobacco seedlings were transferred from soil trays to plastic boxes and pre-cultured for one week in environmentally controlled growth chambers under a condition of 12-h light, 25 °C/12-h dark, 20 °C. The plastic boxes contained a modified Hoagland nutrient solution (4.66 mM Ca(NO_3_)_2_·4H_2_O, 1.41 mM KH_2_PO_4_, 4.98 mM KNO_3_, 1.99 mM MgSO_4_·7H_2_O, 0.10 mM FeSO_4_·7H_2_O, 0.10 mM EDTA-2Na, 46.26 μM H_3_BO_3_, 9.10 μM MnCl_2_·4H_2_O, 0.77 μM ZnCl_2_, 0.41 μM CuCl_2_·2H_2_O, and 0.13 μM Na_2_MoO_4_·2H_2_O) [30].

After pre-culture, tobacco seedlings were transferred to new plastic boxes and were cultured in modified Hoagland nutrient solutions containing four B concentrations (0, 11.57, 46.26, and 185.04 μM), respectively. The nutrient solutions were completely renewed every three days. Each experiment had three replicates (each consists of one plant).

### Measurement of physiological traits

The first fully expanded leaf from the apical meristem of tobacco seedlings grown under different B supply levels was collected at 19 days after treatment (DAT) for measuring physiological traits. The measurements were performed as previously described [30]. Specifically, the contents of Chla, Chlb, and Car were measured using a spectrophotometer following the procedure as described [66]; the contents of RuBPCase, and SP were determined according to the procedures as described by Leech et al. [67], and by Anderson et al. [68], respectively; the activities of AI, NI, NR, SOD, CAT, and POD were measured following the protocols as described [69]; CP was determined by assessing electrolyte leakage as described [70]; and the content of H_2_O_2_ was measured following the procedure as described [71].

### RNA-seq analysis

The first fully expanded leaf was collected from the apical meristem of tobacco seedlings grown under different B supply levels at 5, 12, and 19 DAT, respectively. Three independent biological replicates (each consists of one plant) were used for RNA-seq analysis for each treatment, with each replicate consisting of one plant. The entire leaf was finely ground into powder using liquid nitrogen. Total RNAs were extracted from the ground powders of tobacco leaves using TRIzol reagent (Thermo Fisher Scientific, China), and treated with RNase-free DNaseI (Thermo Fisher Scientific, China) to eliminate genomic DNA contamination. RNA-seq analysis was conducted at Novogene (Beijing, China). The clean data were mapped to the reference genome of tobacco (https://solgenomics.net/organism/Nicotiana_tabacum/genome) [72] using the Hisat2 v2.0.5 program [73]. The expression level of each gene was calculated using the Fragments Per Kilobase of transcript per Million mapped reads (FPKM) value [74]. Differential expression analysis was performed using the DESeq2 program [75]. Genes with a |log2FC|≥ 1 and the false discovery rate (FDR) ≤ 0.05 were considered as significantly differentially expressed.

### Proteome analysis

Total proteins extracted from the first fully expanded leaves of tobacco seedlings grown under different B supply levels collected at 12 DAT according to previously described procedures [76]. The proteins were subjected to TMT-based quantitative proteome analysis [77] at Novogene (Beijing, China). Three independent biological replicates (each consists of one plant) were used for TMT analysis for each treatment.

Briefly, approximately 120 μg total protein of each sample was applied for analysis. The total proteins were digested with trypsin at a trypsin-to-protein mass ratio of 1:50 at 37℃ for 16 h, and then at a ratio of 1:100 for a further 4 h. The digested proteins were labeled using TMT Mass Tagging Kits and Reagents (Thermo Fisher Scientific, USA) following the manufacturer’s instructions. The labeled samples were desalted, lyophilized and fractionated on a Rigol L3000 HPLC system using a C18 column (Waters BEH C18 4.6 × 250 mm, 5 μm). Shotgun proteomics analysis was performed using an EASY-nLCTM 1200 UHPLC system (Thermo Fisher Scientific, USA). The separated peptides were detected using a Q Exactive HF-X mass spectrometer (Thermo Fisher Scientific, USA) with a Nanospray Flex™ ion source.

The obtained spectra were queried against the reference genome (https://solgenomics.net/organism/Nicotiana_tabacum/genome) [72] using the search engines: Proteome Discoverer 2.2. Proteins with a fold change of ≥ 1.2 or ≤ 0.85 and a *P*-value of ≤ 0.05 were determined to be differentially expressed.

### GO analysis

GO enrichment analysis of DEGs and DEPs was performed using the R packages clusterProfiler 4.0 [78]. GO terms with p ≤ 0.05 were considered in this study.

### Quantitative real-time RT-PCR

Total RNAs were extracted from the first fully expanded leaves of tobacco seedling using the TransZol Up kit (TransGen Biotech, China) and were treated with RNase-free DNaseI (Takara, China). First strand cDNAs were synthesized using the HiScript II 1st Strand cDNA Synthesis Kit (Vazyme, China). qRT-PCR was performed on a CFX Connect Real-Time System (BIO-RAD, USA) using a ChamQ Universal SYBR qPCR Master Mix (Vazyme, China). The tobacco *EF-1α* gene [79] was used as the internal control. Three replications were performed for each sample. Primers used for qRT-PCR validation are listed in Table S6.

### Supplementary Information

Supplementary Material 1.

Supplementary Material 2.

Supplementary Material 3.

Supplementary Material 4.

Supplementary Material 5.

Supplementary Material 6.

Supplementary Material 7.

Supplementary Material 8.

## Data Availability

Raw data were deposited in the China National GeneBank DataBase (CNGB) with accession: CNP0004147 (https://db.cngb.org/search/project/CNP0004155).

## Abbreviations

NR: Nitrate reductase
PAL: Phenylalanine ammonia-lyase
PPO: Polyphenoloxidase
AS: Asparagine synthetase
GDH: Glutamate dehydrogenase
DAT: Days after treatment
Chla: Chlorophyll a
Chlb: Chlorophyll b
Car: Carotenoids
SP: Soluble protein
AI: Acid invertase
NI: Neutral invertase
DEGs: Differentially expressed genes
DEPs: Differentially expressed proteins
GO: Gene ontology
BP: Biological process
NIP5;1: Nodulin-26-like intrinsic protein 5;1
BORs: B transporters
EXPs: Expansins
PLLs: Pectate lyase-like proteins
AGPs: Arabinogalactan proteins
FLAs: Fasciclin-like arabinogalactan proteins
XTHs: Xyloglucan endotransglucosylases/hydrolases
PMEs: Pectin methylesterases
PMEIs: Pectin methylesterase inhibitors
C/VIFs: Cell wall/vacuolar inhibitors of fructosidases
INV/PMEI: Invertase/pectin methylesterase inhibitor superfamily proteins
CP: Cell membrane permeability
SODs: Superoxide dismutases
CSDs: Copper/zinc superoxide dismutases
FSDs: Iron superoxide dismutases
CAT2s: Catalases
PODs: Peroxidases
GSTs: Glutathione S-transferases
RG-II: Rhamnogalacturonan-II

## References

[1] O’Neill MA, Ishii T, Albersheim P, Darvill AG. Rhamnogalacturonan II: structure and function of a borate cross-linked cell wall pectic polysaccharide. Annu Rev Plant Biol. 2004;55:109–39. 15377216 10.1146/annurev.arplant.55.031903.141750

[2] Voxeur A, Fry SC. Glycosylinositol phosphorylceramides from Rosa cell cultures are boron-bridged in the plasma membrane and form complexes with rhamnogalacturonan II. Plant J. 2014;79:139–49. 24804932 10.1111/tpj.12547 PMC4230332

[3] Wang N, Yang C, Pan Z, Liu Y, Peng S. Boron deficiency in woody plants: various responses and tolerance mechanisms. Front Plant Sci. 2015;6:916. 26579163 10.3389/fpls.2015.00916 PMC4621400

[4] Eggert K, von Wirén N. Response of the plant hormone network to boron deficiency. New Phytol. 2017;216:868–81. 28833172 10.1111/nph.14731

[5] Landi M, Margaritopoulou T, Papadakis IE, Araniti F. Boron toxicity in higher plants: an update. Planta. 2019;250:1011–32. 31236697 10.1007/s00425-019-03220-4

[6] Brdar-Jokanović M. Boron toxicity and deficiency in agricultural plants. Int J Mol Sci. 2020;21:1424. 32093172 10.3390/ijms21041424 PMC7073067

[7] García-Sánchez F, Simón-Grao S, Martínez-Nicolás JJ, Alfosea-Simón M, Liu C, Chatzissavvidis C, Pérez-Pérez JG, Cámara-Zapata JM. Multiple stresses occurring with boron toxicity and deficiency in plants. J Hazard Mater. 2020;397:122713. 32402955 10.1016/j.jhazmat.2020.122713

[8] Takano J, Wada M, Ludewig U, Schaaf G, von Wirén N, Fujiwara T. The *Arabidopsis* major intrinsic protein NIP5;1 is essential for efficient boron uptake and plant development under boron limitation. Plant Cell. 2006;18:1498–509. 16679457 10.1105/tpc.106.041640 PMC1475503

[9] Routray P, Li T, Yamasaki A, Yoshinari A, Takano J, Choi WG, Sams CE, Roberts DM. Nodulin intrinsic protein 7;1 is a tapetal boric acid channel involved in pollen cell wall formation. Plant Physiol. 2018;178:1269–83. 30266747 10.1104/pp.18.00604 PMC6236609

[10] Tanaka M, Wallace IS, Takano J, Roberts DM, Fujiwara T. NIP6;1 is a boric acid channel for preferential transport of boron to growing shoot tissues in Arabidopsis. Plant Cell. 2008;20:2860–75. 18952773 10.1105/tpc.108.058628 PMC2590723

[11] Poza-Viejo L, Abreu I, González-García MP, Allauca P, Bonilla I, Bolaños L, Reguera M. Boron deficiency inhibits root growth by controlling meristem activity under cytokinin regulation. Plant Cell. 2018;270:176–89. 10.1016/j.plantsci.2018.02.005 29576071

[12] Wu X, Riaz M, Yan L, Du C, Liu Y, Jiang C. Boron deficiency in trifoliate orange induces changes in pectin composition and architecture of components in root cell walls. Front Plant Sci. 2017;8:1882. 29167675 10.3389/fpls.2017.01882 PMC5682329

[13] Nakagawa Y, Hanaoka H, Kobayashi M, Miyoshi K, Miwa K, Fujiwara T. Cell-type specificity of the expression of *OsBOR1*, a rice efflux boron transporter gene, is regulated in response to boron availability for efficient boron uptake and xylem loading. Plant Cell. 2007;19:2624–35. 17675406 10.1105/tpc.106.049015 PMC2002629

[14] Durbak AR, Phillips KA, Pike S, O’Neill MA, Mares J, Gallavotti A, Malcomber ST, Gassmann W, McSteen P. Transport of boron by the tassel-less1 aquaporin is critical for vegetative and reproductive development in maize. Plant Cell. 2014;26:2978–95. 25035406 10.1105/tpc.114.125898 PMC4145126

[15] Hua Y, Zhou T, Ding G, Yang Q, Shi L, Xu F. Physiological, genomic and transcriptional diversity in responses to boron deficiency in rapeseed genotypes. J Exp Bot. 2016;67:5769–84. 27639094 10.1093/jxb/erw342 PMC5066495

[16] Chen L, Xia F, Wang M, Wang W, Mao P. Metabolomic analyses of alfalfa (Medicago sativa L. cv. ’Aohan’) reproductive organs under boron deficiency and surplus conditions. Ecotoxicol Environ Saf. 2020;202:111011. 32800236 10.1016/j.ecoenv.2020.111011

[17] Reid RJ, Hayes JE, Post A, Stangoulis JCR, Graham RD. A critical analysis of the causes of boron toxicity in plants. Plant Cell Environ. 2004;27:1405–14. 10.1111/j.1365-3040.2004.01243.x

[18] Choi EY, Kolesik P, McNeill A, Collins H, Zhang Q, Huynh BL, Graham R, Stangoulis J. The mechanism of boron tolerance for maintenance of root growth in barley (Hordeum vulgare L.). Plant Cell Environ. 2007;30:984–93. 17617826 10.1111/j.1365-3040.2007.01693.x

[19] Aquea F, Federici F, Moscoso C, Vega A, Jullian P, Haseloff J, Arce-Johnson P. A molecular framework for the inhibition of *Arabidopsis* root growth in response to boron toxicity. Plant Cell Environ. 2012;35:719–34. 21988710 10.1111/j.1365-3040.2011.02446.x

[20] Gimeno V, Simόn I, Nieves M, Martínez V, Cámara-Zapata JM, García AL, García-Sánchez F. The physiological and nutritional responses to an excess of boron by Verna lemon trees that were grafted on four contrasting rootstocks. Trees. 2012;26:1513–26. 10.1007/s00468-012-0724-5

[21] Shorrocks VM. The occurrence and correction of boron deficiency. Plant Soil. 1997;193:121–48. 10.1023/A:1004216126069

[22] Li M, Sun H, Sun J, Li J, Zhang X, Zhang K, Wang T, Ji X, Deng X, He C, et al. Moderate boron concentration beneficial for flue-cured tobacco (Nicotiana tabacum L.) seedlings growth and development. Agriculture. 2022;12:1670. 10.3390/agriculture12101670

[23] Li Q, Zhou J, Zhang Y, Tao W, Zhang Z, Xie Y, Liu X. Study on spatial distribution of soil available microelement in Qujing tobacco farming area. China Procedia Environ Sci. 2011;10:185–91. 10.1016/j.proenv.2011.09.032

[24] Camacho-Cristobal JJ, Gonzalez-Fontes A. Boron deficiency causes a drastic decrease in nitrate content and nitrate reductase activity, and increases the content of carbohydrates in leaves from tobacco plants. Planta. 1999;209:528–36. 10550635 10.1007/s004250050757

[25] Camacho-Cristóbal JJ, Anzellotti D, Gonzalez-Fontes A. Changes in phenolic metabolism of tobacco plants during short-term boron deficiency. Plant Physiol Biochem. 2002;40:997–1002. 10.1016/S0981-9428(02)01463-8

[26] Camacho-Cristóbal JJ, Lunar L, Lafont F, Baumert A, Gonzalez-Fontes A. Boron deficiency causes accumulation of chlorogenic acid and caffeoyl polyamine conjugates in tobacco leaves. J Plant Physiol. 2004;161:879–81. 15310078 10.1016/j.jplph.2003.12.003

[27] Camacho-Cristóbal JJ, Maldonado JM, Gonzalez-Fontes A. Boron deficiency increases putrescine levels in tobacco plants. J Plant Physiol. 2005;162:921–8. 16146318 10.1016/j.jplph.2004.09.016

[28] Beato VM, Teresa NM, Rexach J, Begoña HM, Camacho-Cristóbal JJ, Kempa S, Weckwerth W, González-Fontes A. Expression of root glutamate dehydrogenase genes in tobacco plants subjected to boron deprivation. Plant Physiol Biochem. 2011;49:1350–4. 21705226 10.1016/j.plaphy.2011.06.001

[29] Beato VM, Rexach J, Navarro-Gochicoa MT, Camacho-Cristóbal JJ, Herrera-Rodríguez MBA, Maldonado JM, González-Fontes A. A tobacco asparagine synthetase gene responds to carbon and nitrogen status and its root expression is affected under boron stress. Plant Sci. 2010;178:289–98. 10.1016/j.plantsci.2009.12.008

[30] Lu J, Ye R, Qu M, Wang Y, Liang T, Lin J, Xie R, Ke Y, Gao J, Li C, et al. Combined transcriptome and proteome analysis revealed the molecular regulation mechanisms of zinc homeostasis and antioxidant machinery in tobacco in response to different zinc supplies. Plant Physiol Biochem. 2023;202:107919. 37557018 10.1016/j.plaphy.2023.107919

[31] Miwa K, Fujiwara T. Boron transport in plants: co-ordinated regulation of transporters. Ann Bot. 2010;105:1103–8. 20228086 10.1093/aob/mcq044 PMC2887066

[32] Cosgrove DJ. Growth of the plant cell wall. Nat Rev Mol Cell Biol. 2005;6:850–61. 16261190 10.1038/nrm1746

[33] Palusa SG, Golovkin M, Shin SB, Richardson DN, Reddy ASN. Organ-specific, developmental, hormonal and stress regulation of expression of putative pectate lyase genes in *Arabidopsis*. New Phytol. 2007;174:537–50. 17447910 10.1111/j.1469-8137.2007.02033.x

[34] Showalter AM. Arabinogalactan-proteins: structure, expression and function. Cell Mol Life Sci. 2001;58:1399–417. 11693522 10.1007/PL00000784 PMC11337269

[35] Ma Y, Yan C, Li H, Wu W, Liu Y, Wang Y, Chen Q, Ma H. Bioinformatics prediction and evolution analysis of arabinogalactan proteins in the plant kingdom. Front Plant Sci. 2017;8:66. 28184232 10.3389/fpls.2017.00066 PMC5266747

[36] Johnson KL, Jones BJ, Bacic A, Schultz CJ. The fasciclin-like arabinogalactan proteins of Arabidopsis. A multigene family of putative cell adhesion molecules. Plant Physiol. 2003;133:1911–25. 14645732 10.1104/pp.103.031237 PMC300743

[37] Cosgrove DJ. Enzymes and other agents that enhance cell wall extensibility. Annu Rev Plant Physiol Plant Mol Biol. 1999;50:391–417. 11541953 10.1146/annurev.arplant.50.1.391

[38] Campbell P, Braam J. Xyloglucan endotransglycosylases: diversity of genes, enzymes and potential wall-modifying functions. Trends Plant Sci. 1999;4:361–6. 10462769 10.1016/S1360-1385(99)01468-5

[39] Juge N. Plant protein inhibitors of cell wall degrading enzymes. Trends Plant Sci. 2006;11:359–67. 16774842 10.1016/j.tplants.2006.05.006

[40] Coculo D, Lionetti V. The plant invertase/pectin methylesterase inhibitor superfamily. Front Plant Sci. 2022;13:863892. 35401607 10.3389/fpls.2022.863892 PMC8990755

[41] McGonigle B, Keeler SJ, Lau SM, Koeppe MK, O’Keefe DP. A genomics approach to the comprehensive analysis of the glutathione S-transferase gene family in soybean and maize. Plant Physiol. 2000;124:1105–20. 11080288 10.1104/pp.124.3.1105 PMC59210

[42] Onuh AF, Miwa K. Regulation, diversity and evolution of boron transporters in plants. Plant Cell Physiol. 2021;62:590–9. 33570563 10.1093/pcp/pcab025

[43] Wang S, Yoshinari A, Shimada T, Hara-Nishimura I, Mitani-Ueno N, Feng MJ, Naito S, Takano J. Polar localization of the NIP5;1 boric acid channel is maintained by endocytosis and facilitates boron transport in *Arabidopsis* roots. Plant Cell. 2017;29:824–42. 28341806 10.1105/tpc.16.00825 PMC5435427

[44] Zhang D, Hua Y, Wang X, Zhao H, Shi L, Xu F. A high-density genetic map identifies a novel major QTL for boron efficiency in oilseed rape (Brassica napus L.). Plos One. 2014;9:e112089. 25375356 10.1371/journal.pone.0112089 PMC4222981

[45] Leonard A, Holloway B, Guo M, Rupe M, Yu G, Beatty M, Zastrow-Hayes G, Meeley R, Llaca V, Butler K, et al. Tassel-less1 encodes a boron channel protein required for inflorescence development in maize. Plant Cell Physiol. 2014;55:1044–54. 24685595 10.1093/pcp/pcu036

[46] Hanaoka H, Uraguchi S, Takano J, Tanaka M, Fujiwara T. OsNIP3;1, a rice boric acid channel, regulates boron distribution and is essential for growth under boron-deficient conditions. Plant J. 2014;78:890–902. 24654769 10.1111/tpj.12511

[47] Shao JF, Yamaji N, Liu XW, Yokosho K, Shen RF, Ma JF. Preferential distribution of boron to developing tissues is mediated by the intrinsic protein OsNIP3. Plant Physiol. 2018;176:1739–50. 29217595 10.1104/pp.17.01641 PMC5813552

[48] Groszmann M, De Rosa A, Ahmed J, Chaumont F, Evans JR. A consensus on the aquaporin gene family in the allotetraploid plant. Nicotiana Tabacum Plant Direct. 2021;5:e321. 10.1002/pld3.321 PMC8104905 33977216

[49] Granado-Rodríguez S, Bolaños L, Reguera M. MtNIP5;1, a novel *Medicago truncatula* boron diffusion facilitator induced under deficiency. BMC Plant Biol. 2020;20:552. 33297962 10.1186/s12870-020-02750-4 PMC7724820

[50] Feng Y, Cui R, Wang S, He M, Hua Y, Shi L, Ye X, Xu F. Transcription factor BnaA9.WRKY47 contributes to the adaptation of Brassica napus to low boron stress by up-regulating the boric acid channel gene BnaA3.NIP5;1. Plant Biotechnol J. 2020;18:1241–54. 31705705 10.1111/pbi.13288 PMC7152615

[51] Song X, Wang X, Song B, Wu Z, Zhao X, Huang W, Riaz M. Transcriptome analysis reveals the molecular mechanism of boron deficiency tolerance in leaves of boron-efficient *Beta vulgaris* seedlings. Plant Physiol Biochem. 2021;168:294–304. 34670152 10.1016/j.plaphy.2021.10.017

[52] Liu J, Chen T, Wang C, Liu X. Transcriptome analysis in *Pyrus betulaefolia* roots in response to short-term boron deficiency. Genes. 2023;14:817. 37107575 10.3390/genes14040817 PMC10137548

[53] Takano J, Yamagami M, Noguchi K, Hayashi H, Fujiwara T. Preferential translocation of boron to young leaves in *Arabidopsis thaliana* regulated by the *BOR1* gene. Soil Sci Plant Nutr. 2001;47:345–57. 10.1080/00380768.2001.10408398

[54] Takano J, Noguchi K, Yasumori M, Kobayashi M, Gajdos Z, Miwa K, Hayashi H, Yoneyama T, Fujiwara T. *Arabidopsis* boron transporter for xylem loading. Nature. 2002;420:337–40. 12447444 10.1038/nature01139

[55] Miwa K, Wakuta S, Takada S, Ide K, Takano J, Naito S, Omori H, Matsunaga T, Fujiwara T. Roles of BOR2, a boron exporter, in cross linking of rhamnogalacturonan II and root elongation under boron limitation in *Arabidopsis*. Plant Physiol. 2013;163:1699–709. 24114060 10.1104/pp.113.225995 PMC3850200

[56] Miwa K, Aibara I, Fujiwara T. *Arabidopsis thaliana* BOR4 is upregulated under high boron conditions and confers tolerance to high boron. Soil Sci Plant Nutr. 2014;60:349–55. 10.1080/00380768.2013.866524

[57] Zhang B, Gao Y, Zhang L, Zhou Y. The plant cell wall: biosynthesis, construction, and functions. J Integr Plant Biol. 2021;63:251–72. 33325153 10.1111/jipb.13055

[58] Ishii T, Matsunaga T, Hayashi N. Formation of rhamnogalacturonan II-borate dimer in pectin determines cell wall thickness of pumpkin tissue. Plant Physiol. 2001;126:1698–705. 11500567 10.1104/pp.126.4.1698 PMC117168

[59] Liu G, Dong X, Liu L, Wu L, Peng SA, Jiang C. Boron deficiency is correlated with changes in cell wall structure that lead to growth defects in the leaves of navel orange plants. Sci Hortic. 2014;176:54–62. 10.1016/j.scienta.2014.06.036

[60] Camacho-Cristóbal JJ, Herrera-Rodríguez MB, Beato VM, Rexach J, Navarro-Gochicoa MT, Maldonado JM, González-Fontes A. The expression of several cell wall-related genes in *Arabidopsis* roots is down-regulated under boron deficiency. Environ Exp Bot. 2008;63:351–8. 10.1016/j.envexpbot.2007.12.004

[61] Yang L, Qi Y, Lu Y, Guo P, Sang W, Feng H, Zhang H, Chen L. iTRAQ protein profile analysis of *Citrus sinensis* roots in response to long-term boron-deficiency. J Proteomics. 2013;93:179–206. 23628855 10.1016/j.jprot.2013.04.025

[62] Zhou GF, Liu YZ, Sheng O, Wei QJ, Yang CQ, Peng SA. Transcription profiles of boron-deficiency-responsive genes in citrus rootstock root by suppression subtractive hybridization and cDNA microarray. Front Plant Sci. 2015;5:795. 25674093 10.3389/fpls.2014.00795 PMC4309116

[63] Tavanti TR, Melo A, Moreira L, Sanchez D, Silva R, Silva R, Reis A. Micronutrient fertilization enhances ROS scavenging system for alleviation of abiotic stresses in plants. Plant Physiol Biochem. 2021;160:386–96. 33556754 10.1016/j.plaphy.2021.01.040

[64] de Souza JP Jr, de Prado RM, Campos CNS, Sousa Junior GS, Oliveira KR, Cazetta JO, Gratão PL. Addition of silicon to boron foliar spray in cotton plants modulates the antioxidative system attenuating boron deficiency and toxicity. BMC Plant Biol. 2022;22:338. 35831782 10.1186/s12870-022-03721-7 PMC9281171

[65] Mittler R, Zandalinas SI, Fichman Y, Van Breusegem F. Reactive oxygen species signalling in plant stress responses. Nat Rev Mol Cell Biol. 2022;23:663–79. 35760900 10.1038/s41580-022-00499-2

[66] Lichtenthaler HK, Buschmann C. Chlorophylls and carotenoids: measurement and characterization by UV-VIS spectroscopy. Curr Protoc Food Anal Chem. 2001;55:F4,3.1-F4.3.8.

[67] Leech RM, Leese BM, Jellings AJ. Variation in cellular ribulose-1,5-bisphosphate-carboxylase content in leaves of Triticum genotypes at three levels of ploidy. Planta. 1985;166:259–63. 24241441 10.1007/BF00397357

[68] Anderson MD, Prasad TK, Stewart CR. Changes in isozyme profiles of catalase, peroxidase, and glutathione reductase during acclimation to chilling in mesocotyls of maize seedlings. Plant Physiol. 1995;109:1247–57. 12228666 10.1104/pp.109.4.1247 PMC157657

[69] Zou Q. Plant physiology experiments guide. Beijing: China (in Chinese), China Agriculture Press; 2000.

[70] Palta JP, Stadelmann EJ. Effect of turgor pressure on water permeability of *Allium cepa* epidermis cell membranes. J Membr Biol. 1977;33:231–47. 864689 10.1007/BF01869518

[71] Alexieva V, Sergiev I, Mapelli S, Karanov E. The effect of drought and ultraviolet radiation on growth and stress markers in pea and wheat. Plant Cell Environ. 2001;24:1337–44. 10.1046/j.1365-3040.2001.00778.x

[72] Edwards KD, Fernandez-Pozo N, Drake-Stowe K, Humphry M, Evans AD, Bombarely A, Allen F, Hurst R, White B, Kernodle SP, et al. A reference genome for *Nicotiana tabacum* enables map-based cloning of homeologous loci implicated in nitrogen utilization efficiency. BMC Genomics. 2017;18:448. 28625162 10.1186/s12864-017-3791-6 PMC5474855

[73] Kim D, Langmead B, Salzberg SL. HISAT: a fast spliced aligner with low memory requirements. Nat Methods. 2015;12:357–60. 25751142 10.1038/nmeth.3317 PMC4655817

[74] Trapnell C, Williams BA, Pertea G, Mortazavi A, Kwan G, van Baren MJ, Salzberg SL, Wold BJ, Pachter L. Transcript assembly and quantification by RNA-Seq reveals unannotated transcripts and isoform switching during cell differentiation. Nat Biotechnol. 2010;28:511–5. 20436464 10.1038/nbt.1621 PMC3146043

[75] Love MI, Huber W, Anders S. Moderated estimation of fold change and dispersion for RNA-seq data with DESeq2. Genome Biol. 2014;15:550. 25516281 10.1186/s13059-014-0550-8 PMC4302049

[76] Yang LM, Tian DG, Todd CD, Luo YM, Hu XY. Comparative proteome analyses reveal that nitric oxide is an important signal molecule in the response of rice to aluminum toxicity. J Proteome Res. 2013;12:1316–30. 23327584 10.1021/pr300971n

[77] Ross PL, Huang YN, Marchese JN, Williamson B, Parker K, Hattan S, Khainovski N, Pillai S, Dey S, Daniels S, et al. Multiplexed protein quantitation in *Saccharomyces cerevisiae* using amine-reactive isobaric tagging reagents. Mol Cell Proteomics. 2004;3:1154–69. 15385600 10.1074/mcp.M400129-MCP200

[78] Wu T, Hu E, Xu S, Chen M, Guo P, Dai Z, Feng T, Zhou L, Tang W, Zhan L, et al. clusterProfiler 4.0: a universal enrichment tool for interpreting omics data. Innovation (Camb). 2021;2:100141. 34557778 10.1016/j.xinn.2021.100141 PMC8454663

[79] Schmidt GW, Delaney SK. Stable internal reference genes for normalization of real-time RT-PCR in tobacco (*Nicotiana tabacum*) during development and abiotic stress. Mol Genet Genomics. 2010;283:233–41. 20098998 10.1007/s00438-010-0511-1

