# Supplementary material for: Integrative analysis of the transcriptome and proteome reveals the molecular responses of tobacco to boron deficiency
Source: BMC Plant Biol. 2024 Jul 19;24:689. doi: 10.1186/s12870-024-05391-z (PMC11264865; doi:10.1186/s12870-024-05391-z)
Supplement: Supplementary file 6 — Supplementary Material 6. [file 12870_2024_5391_MOESM6_ESM.docx]

**Table S6. Primers used for qRT-PCR validation**

| **Primer** | **Sequence (5ʹ-3ʹ)** | **Comment** |
| --- | --- | --- |
| NIP5;1(1)-F | TACTATTGCGTGCTTGAA | F and R primers for qRT-PCR analysis of *NIP5;1* (*Nitab4.5_0000799g0080*) |
| NIP5;1(1)-R | CATAGCGAATGAGGAGAA |  |
| NIP5;1(2)-F | TGCTGAAGGAAGGATGTT | F and R primers for qRT-PCR analysis of *NIP5;1* (*Nitab4.5_0005519g0010*) |
| NIP5;1(2)-R | TTGGTAGTGAGACTGGAAG |  |
| EXPA3-F | ACAGTGGATAGCAATGAG | F and R primers for qRT-PCR analysis of *EXPA3* (*Nitab4.5_0003845g0060*) |
| EXPA3-R | CTTTAACCCTAATGGACAAG |  |
| EXPA14-F | GCTCATATCTCACCACTCTC | F and R primers for qRT-PCR analysis of *EXPA14* (*Nitab4.5_0004599g0080*) |
| EXPA14-R | GCTCATATCTCACCACTCTC |  |
| PLL12-F | GTCCACTGCTATTACTAT | F and R primers for qRT-PCR analysis of *PLL12* (*Nitab4.5_0001359g0050*) |
| PLL12-R | GTCCACTGCTATTACTAT |  |
| PLL25-F | TTGTTGATGCTGTTATGG | F and R primers for qRT-PCR analysis of *PLL25* (*Nitab4.5_0010267g0020*) |
| PLL25-R | TGCTTGTCTCTAGTGTAG |  |
| FSD2-F | TGAGCCATACTACATTCG | F and R primers for qRT-PCR analysis of *FSD2* (*Nitab4.5_0000103g0130*) |
| FSD2-R | GTGTCATTCCATCCAGTT |  |
| CAT2-F | CTCATTCCAGTCATATAGCAAGAT | F and R primers for qRT-PCR analysis of *CAT2* (*Nitab4.5_0000702g0130*) |
| CAT2-R | CATTCATTCATACAACAGCAACA |  |
| POD52-F | GATGGAGGAGTTCTTCTA | F and R primers for qRT-PCR analysis of *POD52* (*Nitab4.5_0015635g0010*) |
| POD52-R | GCTTGTTCTATGACTTCA |  |
| EF-1α-F | TGAGATGCACCACGAAGCTC | F and R primers for qRT-PCR analysis of the internal control *NtEF-1*α |
| EF-1α-R | CCAACATTGTCACCAGGAAGTG |  |
