# Supplementary material for: Integrative analysis of the transcriptome and proteome reveals the molecular responses of tobacco to boron deficiency
Source: BMC Plant Biol. 2024 Jul 19;24:689. doi: 10.1186/s12870-024-05391-z (PMC11264865; doi:10.1186/s12870-024-05391-z)
Supplement: Supplementary file 7 — Supplementary Material 7. [file 12870_2024_5391_MOESM7_ESM.pptx]

## Slide 1
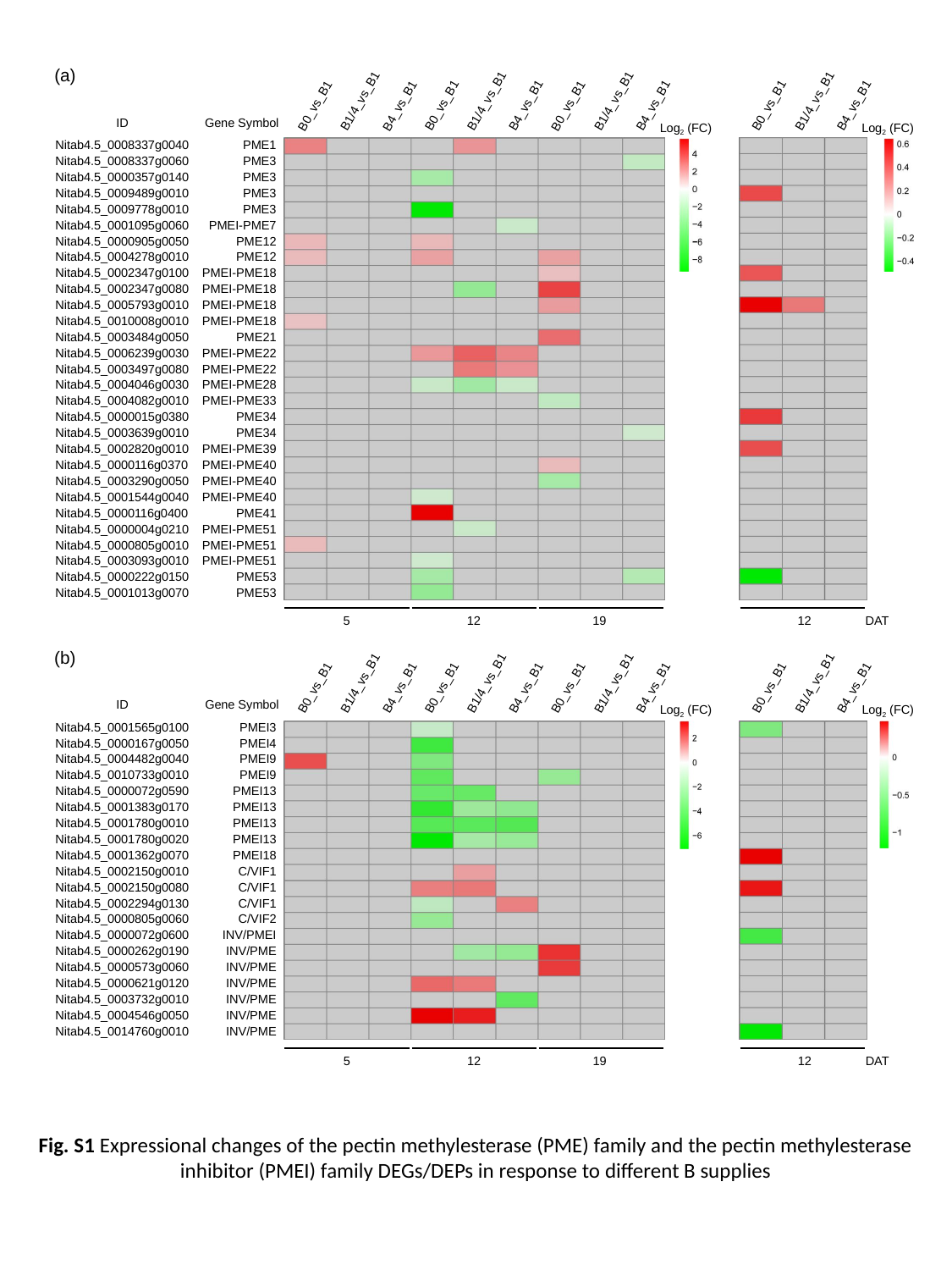

(a)
B1/4_vs_B1
B1/4_vs_B1
B1/4_vs_B1
B1/4_vs_B1
B0_vs_B1
B4_vs_B1
B0_vs_B1
B4_vs_B1
B0_vs_B1
B4_vs_B1
B0_vs_B1
B4_vs_B1
Gene Symbol
ID
Log2 (FC)
Log2 (FC)
| Nitab4.5\_0008337g0040 | PME1 |
| --- | --- |
| Nitab4.5\_0008337g0060 | PME3 |
| Nitab4.5\_0000357g0140 | PME3 |
| Nitab4.5\_0009489g0010 | PME3 |
| Nitab4.5\_0009778g0010 | PME3 |
| Nitab4.5\_0001095g0060 | PMEI-PME7 |
| Nitab4.5\_0000905g0050 | PME12 |
| Nitab4.5\_0004278g0010 | PME12 |
| Nitab4.5\_0002347g0100 | PMEI-PME18 |
| Nitab4.5\_0002347g0080 | PMEI-PME18 |
| Nitab4.5\_0005793g0010 | PMEI-PME18 |
| Nitab4.5\_0010008g0010 | PMEI-PME18 |
| Nitab4.5\_0003484g0050 | PME21 |
| Nitab4.5\_0006239g0030 | PMEI-PME22 |
| Nitab4.5\_0003497g0080 | PMEI-PME22 |
| Nitab4.5\_0004046g0030 | PMEI-PME28 |
| Nitab4.5\_0004082g0010 | PMEI-PME33 |
| Nitab4.5\_0000015g0380 | PME34 |
| Nitab4.5\_0003639g0010 | PME34 |
| Nitab4.5\_0002820g0010 | PMEI-PME39 |
| Nitab4.5\_0000116g0370 | PMEI-PME40 |
| Nitab4.5\_0003290g0050 | PMEI-PME40 |
| Nitab4.5\_0001544g0040 | PMEI-PME40 |
| Nitab4.5\_0000116g0400 | PME41 |
| Nitab4.5\_0000004g0210 | PMEI-PME51 |
| Nitab4.5\_0000805g0010 | PMEI-PME51 |
| Nitab4.5\_0003093g0010 | PMEI-PME51 |
| Nitab4.5\_0000222g0150 | PME53 |
| Nitab4.5\_0001013g0070 | PME53 |
5
12
19
12 DAT
(b)
B1/4_vs_B1
B1/4_vs_B1
B1/4_vs_B1
B1/4_vs_B1
B0_vs_B1
B4_vs_B1
B0_vs_B1
B4_vs_B1
B0_vs_B1
B4_vs_B1
B0_vs_B1
B4_vs_B1
Gene Symbol
ID
Log2 (FC)
Log2 (FC)
| Nitab4.5\_0001565g0100 | PMEI3 |
| --- | --- |
| Nitab4.5\_0000167g0050 | PMEI4 |
| Nitab4.5\_0004482g0040 | PMEI9 |
| Nitab4.5\_0010733g0010 | PMEI9 |
| Nitab4.5\_0000072g0590 | PMEI13 |
| Nitab4.5\_0001383g0170 | PMEI13 |
| Nitab4.5\_0001780g0010 | PMEI13 |
| Nitab4.5\_0001780g0020 | PMEI13 |
| Nitab4.5\_0001362g0070 | PMEI18 |
| Nitab4.5\_0002150g0010 | C/VIF1 |
| Nitab4.5\_0002150g0080 | C/VIF1 |
| Nitab4.5\_0002294g0130 | C/VIF1 |
| Nitab4.5\_0000805g0060 | C/VIF2 |
| Nitab4.5\_0000072g0600 | INV/PMEI |
| Nitab4.5\_0000262g0190 | INV/PME |
| Nitab4.5\_0000573g0060 | INV/PME |
| Nitab4.5\_0000621g0120 | INV/PME |
| Nitab4.5\_0003732g0010 | INV/PME |
| Nitab4.5\_0004546g0050 | INV/PME |
| Nitab4.5\_0014760g0010 | INV/PME |
5
12
19
12 DAT
Fig. S1 Expressional changes of the pectin methylesterase (PME) family and the pectin methylesterase inhibitor (PMEI) family DEGs/DEPs in response to different B supplies
