# Supplementary material for: Integrative analysis of the transcriptome and proteome reveals the molecular responses of tobacco to boron deficiency
Source: BMC Plant Biol. 2024 Jul 19;24:689. doi: 10.1186/s12870-024-05391-z (PMC11264865; doi:10.1186/s12870-024-05391-z)
Supplement: Supplementary file 8 — Supplementary Material 8. [file 12870_2024_5391_MOESM8_ESM.pptx]

## Slide 1
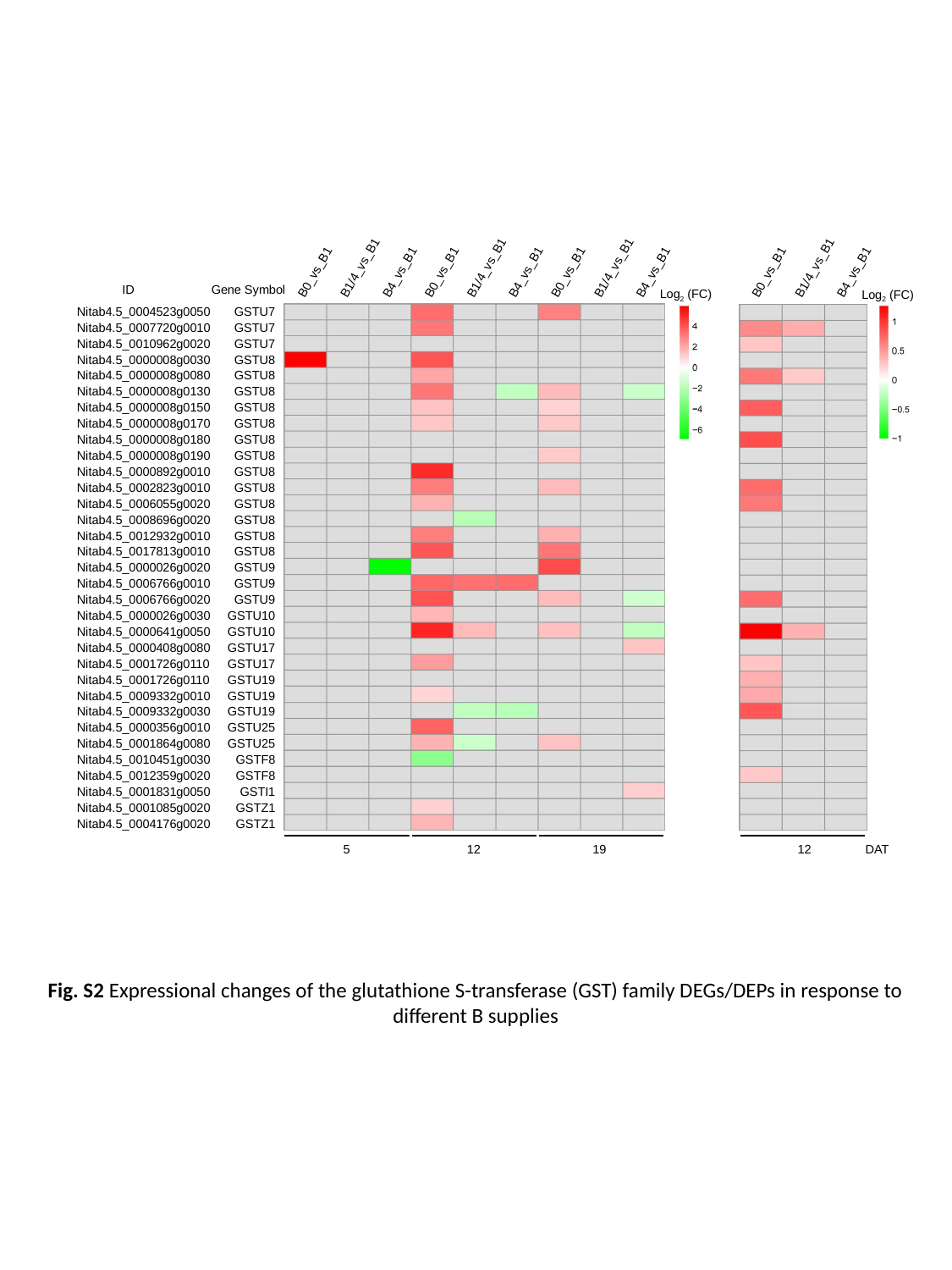

B1/4_vs_B1
B1/4_vs_B1
B1/4_vs_B1
B1/4_vs_B1
B0_vs_B1
B4_vs_B1
B0_vs_B1
B4_vs_B1
B0_vs_B1
B4_vs_B1
B0_vs_B1
B4_vs_B1
Gene Symbol
ID
Log2 (FC)
Log2 (FC)
| Nitab4.5\_0004523g0050 | GSTU7 |
| --- | --- |
| Nitab4.5\_0007720g0010 | GSTU7 |
| Nitab4.5\_0010962g0020 | GSTU7 |
| Nitab4.5\_0000008g0030 | GSTU8 |
| Nitab4.5\_0000008g0080 | GSTU8 |
| Nitab4.5\_0000008g0130 | GSTU8 |
| Nitab4.5\_0000008g0150 | GSTU8 |
| Nitab4.5\_0000008g0170 | GSTU8 |
| Nitab4.5\_0000008g0180 | GSTU8 |
| Nitab4.5\_0000008g0190 | GSTU8 |
| Nitab4.5\_0000892g0010 | GSTU8 |
| Nitab4.5\_0002823g0010 | GSTU8 |
| Nitab4.5\_0006055g0020 | GSTU8 |
| Nitab4.5\_0008696g0020 | GSTU8 |
| Nitab4.5\_0012932g0010 | GSTU8 |
| Nitab4.5\_0017813g0010 | GSTU8 |
| Nitab4.5\_0000026g0020 | GSTU9 |
| Nitab4.5\_0006766g0010 | GSTU9 |
| Nitab4.5\_0006766g0020 | GSTU9 |
| Nitab4.5\_0000026g0030 | GSTU10 |
| Nitab4.5\_0000641g0050 | GSTU10 |
| Nitab4.5\_0000408g0080 | GSTU17 |
| Nitab4.5\_0001726g0110 | GSTU17 |
| Nitab4.5\_0001726g0110 | GSTU19 |
| Nitab4.5\_0009332g0010 | GSTU19 |
| Nitab4.5\_0009332g0030 | GSTU19 |
| Nitab4.5\_0000356g0010 | GSTU25 |
| Nitab4.5\_0001864g0080 | GSTU25 |
| Nitab4.5\_0010451g0030 | GSTF8 |
| Nitab4.5\_0012359g0020 | GSTF8 |
| Nitab4.5\_0001831g0050 | GSTI1 |
| Nitab4.5\_0001085g0020 | GSTZ1 |
| Nitab4.5\_0004176g0020 | GSTZ1 |
5
12
19
12 DAT
Fig. S2 Expressional changes of the glutathione S-transferase (GST) family DEGs/DEPs in response to different B supplies
